# Drug survival of biologics in treating psoriasis: a meta-analysis of real-world evidence

**DOI:** 10.1038/s41598-018-34293-y

**Published:** 2018-10-30

**Authors:** Pei-Tzu Lin, Shu-Hui Wang, Ching-Chi Chi

**Affiliations:** 1 Department of Pharmacy, Chang Gung Memorial Hospital, Chiayi, Chiayi, Taiwan; 2 Department of Nursing, Chang Gung University of Science and Technology, Chiayi, Taiwan; 3 Department of Dermatology, Far Eastern Memorial Hospital, New Taipei, Taiwan; 4 Graduate Institute of Applied Science and Engineering, College of Science and Engineering, Fu Jen Catholic University, New Taipei, Taiwan; 5 Department of Dermatology, Chang Gung Memorial Hospital, Linkou, Taoyuan, Taiwan; 6 College of Medicine, Chang Gung University, Taoyuan, Taiwan

## Abstract

Drug survival of biologics represents their real-world effectiveness and safety. We conducted a meta-analysis of real-world evidence on the drug survival of biologics in treating psoriasis. We searched the PubMed, CENTRAL, and EMBASE databases from inception to 7th October 2017 for studies reporting the annual drug survival for at least 1 year. Two authors independently screened and selected relevant studies, and assessed their risk of bias. A third author was available for arbitrating discrepancies. We conducted a random-effects model meta-analysis to obtain the respective pooled drug survival from year 1 to 4. We conducted subgroup analysis on biologic-naïve subjects, discontinuation for loss of efficacy and adverse effects. We included 37 studies with 32,631 subjects. The drug survival for all biologics decreased with time, dropping from 66% at year 1 to 41% at year 4 for etanercept, from 69% to 47% for adalimumab, from 61% to 42% for infliximab, and from 82% to 56% for ustekinumab. Ustekinumab was associated with the highest drug survival in all and biologic-naïve subjects. Etanercept was associated with the lowest drug survival and was most commonly discontinued for loss of efficacy. Infliximab was most frequently associated with discontinuation for adverse effects. Clinicians may use this study as a reference in treating psoriasis.

## Introduction

Psoriasis is a chronic inflammatory dermatosis^1,2^. Biologics are indicated for treating moderate to severe psoriasis that is unresponsive to conventional systemic therapies or phototherapy or when the patients are intolerant to traditional treatments^3,4^. A recent network meta-analysis has demonstrated excellent short-term efficacy of biologics, including tumor necrosis factor inhibitors (for example etanercept, infliximab, and adalimumab), interleukin (IL)−12/23 inhibitors (such as ustekinumab), IL-17 inhibitors (including secukinumab, ixekizumab, and brodalumab), and IL-23 inhibitors (such as guselkumab and tildrakizumab)^5^. However, the efficacy of biologics in treating psoriasis usually fades with time. Drug survival, also known as ‘drug retention’ or ‘drug persistence’, is the rate and duration of adherence to biologics, which represent the long-term effectiveness and safety of the biologics in the real world. Drug survival is a useful reference for choosing a biologic for treating psoriasis.

To date there are only individual studies on the drug survival of biologics for treating psoriasis. No *et al*.^6^ conducted a systemic review of biologic agents for psoriasis and revealed ustekinumab was associated with the longest median drug survival (38.0 months), which was longer than those of infliximab, adalimumab, and etanercept. However, they did not assess the safety data nor analyze the drug survival in biologic-naïve subjects. To provide physicians with a reference for administering biologics for various clinical settings, we conducted a systematic review and meta-analysis of real-world evidence on the drug survival of biologics in treating psoriasis, and conducted subgroup analyses on biologic-naïve subjects, discontinuation due to loss of efficacy and adverse effects.

## Results

### Identification and characteristics of studies

**Figure 1:**
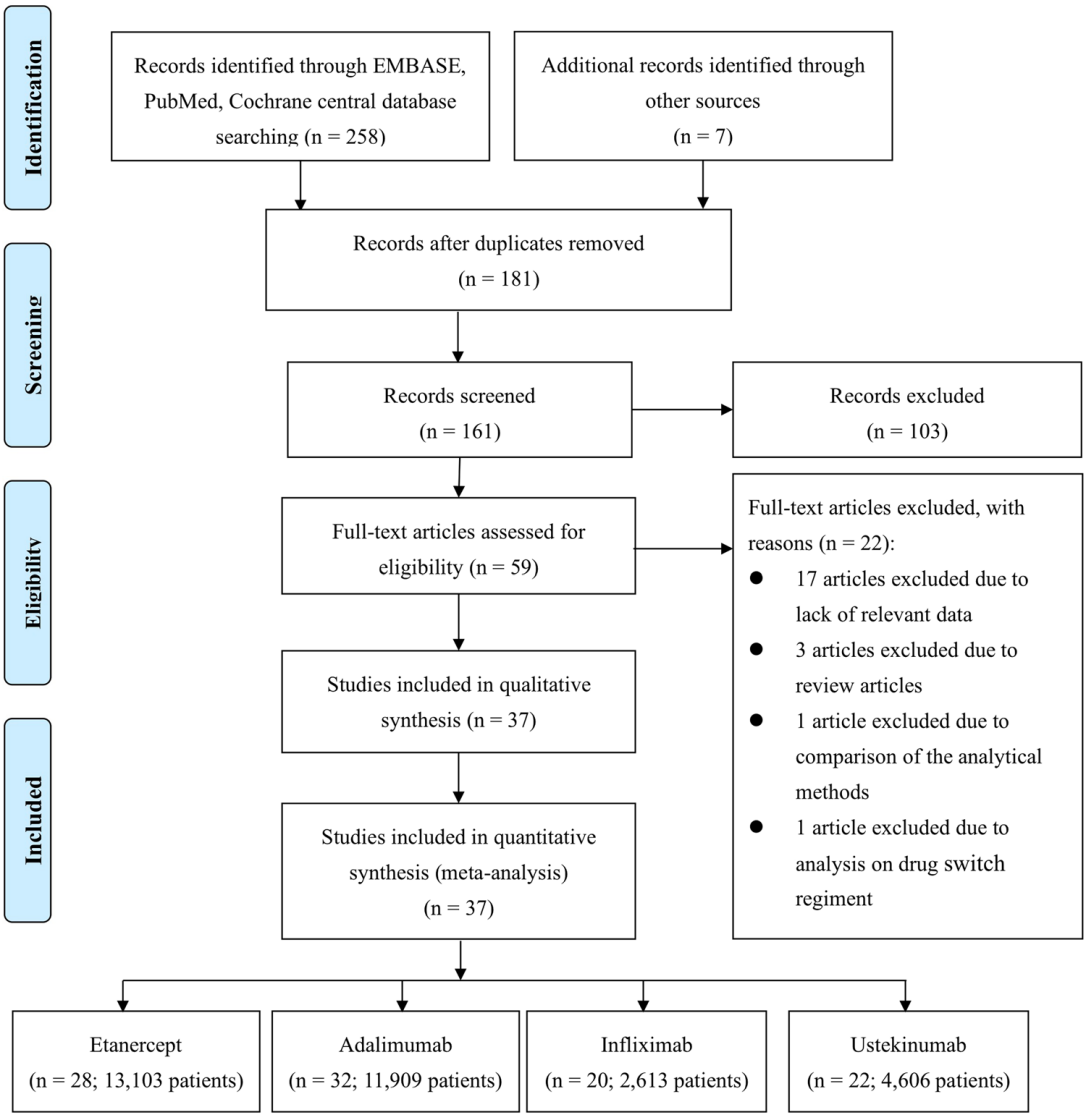
The PRISMA study flow diagram. Adapted from the PRISMA template^7^.

The PRISMA^7^ study flow diagram is shown in Fig. 1. Our search identified 181 articles after removing 84 duplicates. A total of 37 studies with 32,231 subjects met our inclusion criteria and were included. Six included studies provided usable data for the meta-analysis on discontinuation due to ineffectiveness, while five included studies were available for the meta-analysis on discontinuation due to adverse effects.

As to the risk of bias, two studies were rated five stars, twelve of the studies were rated six stars, nineteen of the studies were rated seven, and four were rated eight stars on the Newcastle–Ottawa Scale (NOS)^8^.

### Drug survival of biologics for treating psoriasis

The drug survival for each biologic and the status of patients being biologic-naïve or -experienced are shown in Supplementary Table S1. There were 28 studies^9–36^ (13,303 patients) reporting data for etanercept, 32 studies^9–11,13–19,21–31,33–43^ (12,109 patients) for adalimumab, 20 studies^9,11,13–19,21–24,26,27,29,30,34,35,40^ (2,613 patients) for infliximab, and 22 studies^9,13,14,16,19,22–31,33–36,40,44,45^ (4,606 patients) for ustekinumab.

**Figure 2:**
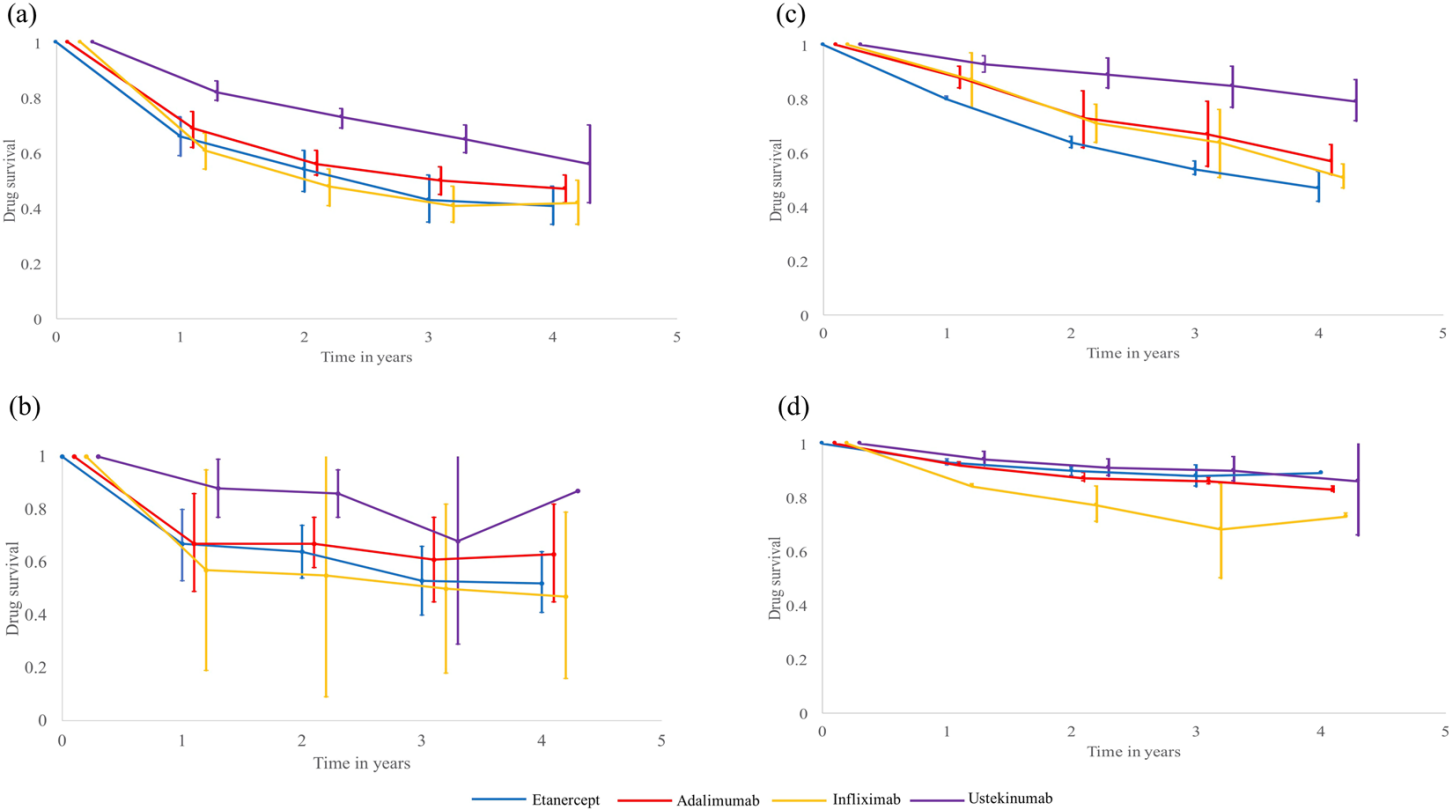
Drug survival of biologics for treating psoriasis in (**a**) all subjects, (**b**) biologic-naïve subjects, and as to (**c**) discontinue due to loss of efficacy and (**d**) adverse effects.

The meta-analysis on the drug survival of each biologic is show in Fig. 2(a), with the forest plots shown in the Supplementary Fig. S1. The drug survival for all biologics decreased year by year. The drug survival rate for etanercept was 1 (i.e. 100%) at year 0 (baseline) and dropped to 0.66 (95% confidence interval (CI) 0.59‒0.73) at year 1, and 0.41 (95% CI 0.34‒0.48) at year 4. That is, of 100 patients on etanercept therapy at baseline, on average 66 remained on etanercept therapy 1 year later, and 41 remained on the same therapy 4 years later. The drug survival rate dropped from 0.69 (95% CI 0.62‒0.75) at year 1 to 0.47 (95% CI 0.42‒0.52) at year 4 for adalimumab, from 0.61 (95% CI 0.54‒0.67) to 0.42 (95% CI 0.34‒0.50) for infliximab, and from 0.82 (95% CI 0.79‒0.86) to 0.56 (95% CI 0.42‒0.70) for ustekinumab. Overall, etanercept was associated with the lowest drug survival rate and ustekinumab associated with the highest one.

### Subgroup analysis on biologic-naïve subjects

The subgroup analysis limited to biologic-naïve subjects is illustrated in Fig. 2(b), with the forest plots shown in Supplementary Fig. S2. There were 7 studies (2,536 patients) reporting data on biologic-naïve patients for etanercept, 7 studies (2,455 patients) for adalimumab, 4 studies (136 patients) for infliximab, and 4 studies (368 patients) for ustekinumab. The drug survival in biologic-naïve patients was generally higher than that in all subjects. The drug survival in biologic-naïve subjects gradually decreased with time for all biologics, too. The drug survival rate fell from 0.67 (95% CI 0.53‒0.80) at year 1 to 0.52 (95% CI 0.41‒0.64) at year 4 for etanercept, from 0.67 (95% CI 0.49‒0.86) to 0.63 (95% CI 0.45‒0.82) for adalimumab, from 0.57 (95% CI 0.19‒0.95) to 0.47 (95% CI 0.16‒0.79) for infliximab, and from 0.88 (95% CI 0.77‒0.99) to 0.87 (95% CI 0.87‒0.87) for ustekinumab. Ustekinumab had a trough drug survival at year 3 because the study of Mitratza *et al*.^26^. Contributed data to the low survival at year 3. In general, ustekinumab was associated with the highest drug survival rate and infliximab associated with the lowest rate in biologic-naïve subjects.

### Discontinuation of biologics due to loss of efficacy

The data on discontinuation due to loss of efficacy are shown in Table 1. There were 5 studies (2,338 patients) reporting data for etanercept, 6 studies (3,580 patients) for adalimumab, 3 studies (368patients) for infliximab, and 5 studies (1,249 patients) for ustekinumab. The meta-analysis on drug survival as to discontinuation due to loss of efficacy from year 1 to 4 is shown as Fig. 2(c) and forest plot is show in Supplementary Fig. S3. The drug survival rate fell from 0.80 (95% CI 0.80‒0.81) at year 1 to 0.47 (95% CI 0.42‒0.53) at year 4 for etanercept, from 0.88 (95% CI 0.84‒0.92) to 0.57 (95% CI 0.52‒0.53) for adalimumab, from 0.87 (95% CI 0.77‒0.97) to 0.51 (95% CI 0.47‒0.56) for infliximab, and from 0.93 (95% CI 0.90‒0.99) to 0.79 (95% CI 0.72‒0.87) for ustekinumab. Etanercept was the biologics most commonly associated with discontinuation due to loss of efficacy and ustekinumab was the least one.

**Table 1:** Studies reporting discontinuation of biologics due to loss of efficacy or adverse effects.

References	Patient papulation	Drug survival rate													
		1 year		2 years		3 years		4 years		5 years		6 years		10 years	
		n	%	n	%	n	%	n	%	n	%	n	%	n	%
**Loss of efficacy**															
Etanercept															
Zweegers *et al*.^36^	238	193	81.1	161	67.6	148	62.2	122	51.4	107	45	—	—	74	31
Vilarrasa *et al*.^34^	248	192	77.6	142	57.1	116	46.9	101	40.8	—	—	—	—	—	—
Warren *et al*.^35^	1098	878	80	714	65	604	55	—	—	—	—	—	—	—	—
Van den Reek *et al*.^31^	82	68	83	—	—	—	—	—	—	—	—	—	—	—	—
Davila-Seijo *et al*.^13^	672	543	80.8	439	65.4	362	53.9	336	50.0						
Adalimumab															
Zweegers *et al*.^36^	186	161	86.5	136	73.0	121	64.9	116	62.2	100	54	93	50		
Vilarrasa *et al*.^34^	231	188	81.6	155	67.3	141	61.2	118	51	—	—	—	—	—	—
Van den Reek *et al*.^41^	459	—	—	—	—	—	—	285	62	—	—	—	—	—	—
Warren *et al*.^35^	1879	1691	90	1560	83	1484	79	—	—	—	—	—	—	—	—
Van den Reek *et al*.^31^	101	86	85	—	—	—	—	—	—	—	—	—	—	—	—
Davila-Seijo *et al*.^13^	724	696	96.2	487	67.3	446	61.5	390	53.9						
Infliximab															
Vilarrasa *et al*.^34^	84	67	79.6	55	65.3	48	57.1	41	49	—	—	—	—	—	—
Warren *et al*.^35^	96	83	86	76	79	73	76	—	—	—	—	—	—	—	—
Davila-Seijo *et al*.^13^	188	181	96.2	130	69.2	108	57.7	101	53.9						
Ustekinumab															
Zweegers *et al*.^36^	102	94	91.9	88	86.5	88	86.5	85	83.8	81	79	—	—	—	—
Vilarrasa *et al*.^34^	140	131	93.8	131	93.8	120	85.7	114	81.6	—	—	—	—	—	—
Warren *et al*.^35^	450	432	96	419	93	401	89	—	—	—	—	—	—	—	—
Van den Reek *et al*.^31^	66	62	94	—	—	—	—	—	—	—	—	—	—	—	—
Davila-Seijo *et al*.^13^	491	444	90.4	415	84.6	378	76.9	359	73.1						
**Adverse effects**															
Etanercept															
Zweegers *et al*.^36^	238	228	95.9	212	89.2	212	89.2	212	89.2	—	—	—	—	167	70
Warren *et al*.^35^	1098	1032	94.0	999	91.0	988	90.0	—	—	—	—	—	—	—	—
Van den Reek *et al*.^31^	82	74	90.0	—	—	—	—	—	—	—	—	—	—	—	—
Davila-Seijo *et al*.^13^	672	620	92.3	594	88.5	569	84.6	597	88.8						
Adalimumab															
Zweegers *et al*.^36^	186	166	89.2	156	83.8	153	82.4	151	81.1	—	—	130	70	—	—
Van den Reek^41^	459	—	—	—	—	—	—	386	84.0	—	—	—	—	—	—
Warren *et al*.^35^	1879	1747	93.0	1681	90.0	1635	87	—	—	—	—	—	—	—	—
Van den Reek *et al*.^31^	101	96	95.0	—	—	—	—	—	—	—	—	—	—	—	—
Davila-Seijo *et al*.^13^	724	668	92.3	640	88.5	640	88.5	613	84.6						
Infliximab															
Warren *et al*.^35^	96	81	84.0	71	74.0	57	59.0	—	—	—	—	—	—	—	—
Davila-Seijo *et al*.^13^	188	159	84.6	152	80.8	145	76.9	137	73.1						
Ustekinumab															
Zweegers *et al*.^36^	102	94	91.9	85	83.8	85	83.8	77	75.7						
Warren *et al*.^35^	450	432	96.0	419	93.0	410	91.0	—	—	—	—	—	—	—	—
Van den Reek *et al*.^31^	66	63	95.0	—	—	—	—	—	—	—	—	—	—	—	—
Davila-Seijo *et al*.^13^	491	491	100.0	472	96.2	472	96.2	472	96.2						

### Discontinuation of biologics due to adverse effects

The data on discontinuation due to adverse effects are reported in Table 1. There were 4 studies (2,090 patients) providing data for etanercept, 5 studies (3,349 patients) for adalimumab, 2 studies (284 patients) for infliximab, and 4 studies (1,109 patients) for ustekinumab. The meta-analysis on the drug survival as to discontinuation due to adverse effects is illustrated in Fig. 2(d) and the forest plots are shown in Supplementary Fig. S4. Ustekinumab was the least frequently associated with discontinuation due to adverse effects. On the other hand, infliximab was most frequently associated with discontinuation due to adverse effects.

## Discussion

### Main findings

To the best of our knowledge, this study is the first to conduct a meta-analysis on the drug survival of biologics for treating psoriasis. We found ustekinumab had the best drug survival in all subjects (including biologic-naïve and experienced ones) and had significantly higher drug survival than the other biologics in the first three years. Ustekinumab had the highest drug survival in biologic-naïve subjects, but did not have a significantly higher drug survival than the other biologics. Etanercept had the worst drug survival in the primary analysis on all subjects; but when limited to biologic-naïve patients, infliximab had the worst drug survival. Etanercept was most often associated with discontinuation due to loss of efficacy, while ustekinumab was the least common. Ustekinumab were the least likely to be discontinued due to adverse effects. Infliximab were the most common biologics discontinued due to adverse effects.

### Strengths and limitations

Our study has several strengths. Firstly, this study included 37 studies with 32,231 subjects, and the average number of study subjects for each biologic was 8,158 patients. Thus, a large sample was available for this meta-analysis on the drug survival. Secondly, the available data enabled us to conduct subgroup analysis on biologic-naïve subjects, discontinuation due to loss of efficacy and adverse effects. Therefore, our study provides drug survival data for various clinical scenarios in the real world. Thirdly, we did not impose any language limitations and thus increased the scope of data searched and collected.

On the other hand, our study had some limitations. Firstly, the study population of included studies were mainly from Europe and thus contained very few Asian patients. Secondly, those studies were observational and might have biases to influence the drug survival, such as selection bias due to non-random allocation. However, randomized trials on drug survival are unlikely to be conducted by pharmaceutical companies. Thirdly, other patients’ baseline characteristics, such as patients’ body weight, age, gender, comorbidities, and co-treatments for psoriasis, might have influenced the drug survival which was reflected by high statistical heterogeneity. However, we could not adjust these factors in our analysis because the included studies did not have relevant details, except for one study^36^ showing a high body mass index and female sex might have affected the drug survival. Fourthly, drug survival could be a surrogate of treatment efficacy and safety to aid clinical making decision. Although we performed subgroup analyses on discontinuation due to loss of efficacy and adverse effects which may be a useful reference for clinical practice, the available sample size was limited. Fifthly, we did not have adequate data on drug survival of biologics newly available on the market, for example secukinumab and ixekizumab. Sixthly, due to the lack of information on costs and insurance plan for each study subjects, we could not examine their effects on drug survival. For example, people in the US can change their insurance plan. However, there was only one US study included in our analysis^14^.

### Implications for practice

The current evidence suggests that ustekinumab is associated with the best drug survival in all and biologic-naïve subjects. Also, ustekinumab is the biologic least frequently associated with discontinuation due to loss of efficacy. Adalimumab has the second-best drug survival. Etanercept has the worst overall drug survival and is the biologic most commonly discontinued due to loss of efficacy. Infliximab has the lowest drug survival among biologic-naïve subjects.

No *et al*.^6^. conducted a systematic review including 21 studies with 20,297 subjects, which was smaller than our study. They reported the median drug survival for ustekinumab, infliximab, adalimumab and etanercept were 38.0 months, 36.5 months, 26.6 months, and 24.7 months, separately. Although they claimed ustekinumab had the highest drug survival, there were no significant differences between ustekinumab and other biologics as the interquartile range of their median drug survival overlapped. They found the drug survival gradually decreased and ustekinumab had the best drug survival, which were in congruent with our study. The study of No *et al*.^6^ only included studies published in English with at least 50 subjects aged at least 18 years; by contrast, our study had no limitations in language, participant characteristics, and sample size. The difference in the inclusion and exclusion criteria of the two studies might have accounted for the discrepancy in the biologic with the second-best drug survival.

The included drug survival studies did not consider the costs of treatments. One previous study on the cost-efficacy of biologics for treating psoriasis demonstrates ustekinumab and adalimumab had the lowest 1-year cost per PASI 75 responder, while ustekinumab had the lowest 2-year cost per PASI 75 responder^46^. Clinicians may consider the data on drug survival and cost efficacy in choosing biologics for treating psoriasis to achieve clinical benefit at the least medical expenditure.

## Conclusions

Drug survival represents the long-term effectiveness and safety in the real-world setting, and is a useful reference for clinical practice. The current evidence suggests ustekinumab is the preferred biologics for treating psoriasis. Further studies are required to complement the current limited data on discontinuation due to loss of efficacy and adverse effects. Clinicians may consider the data on drug survival in choosing biologics in treating psoriasis.

## Methods

### Identification of studies and data extraction

We searched the PubMed, Cochrane Central Register of Controlled Trials (CENTRAL), and EMBASE databases from inception to 7th October 2017 for relevant studies. To increase the sensitivity of search, we also used snowball method, i.e. we scanned the references list of included studies to see if there were other relevant studies. We used the following terms in searching electronic databases: “drug retention” or “drug survival” or “drug persistence” and “psoriasis”. Studies that analyzed the drug survival of biologics and reported the respective annual data for each biologic for at least 1 year were included. Studies that did not report the respective survival of biologics or did not have outcome data for at least 1 year were excluded.

We scanned the titles and abstracts of the search results and obtained the full text of potentially eligible studies for further evaluation. Two investigators (PL and CC) independently screened, selected relevant studies, and used the NOS^8^ to assess the risk of bias of included studies. A third author (SW) was available for arbitration when discrepancy occurred. We did not impose any language restrictions.

### Risk of bias assessment

The NOS uses three categories (selection of study groups, comparability, and outcome assessment) to evaluate the risk of bias of cohort studies. As to the selection category, four items are evaluated, including (1) the representativeness of exposed cohort, (2) selection of non-exposed cohort, (3) ascertainment of exposure, and (4) outcome of the interest not present at start of study. Regarding the comparability category, the comparability of cohorts was assessed. In the outcome category, two items were rated: (1) follow-up duration and (2) adequacy of follow up of cohorts. A study could be awarded up to one star for each numbered item within the selection and outcome categories and up to two stars for comparability. The highest quality studies are awarded up to nine stars^8^.

### Data extraction

We used a pre-determined data extraction form to collect data including the first author’s last name, publication year, type of biologics, a history of receiving any biologics before or not (biologic-naïve or -experienced status), sample size, survival (percentage or number of patients) per year (from year 1 to 4), and the respective rate of withdrawal due to loss of efficacy and adverse effects. We did not extract data of efalizumab which has been withdrawn from the market.

### Statistical analysis

The drug survival rate and numbers of patients of each biologic in each study were collected. As we expected clinical heterogeneity across the included studies, we implemented a random-effects model meta-analysis using the generic inverse variance method to calculate the pooled drug survival rate of each biologic. The drug survival rate was expressed as mean difference (MD) with 95% confidence interval (CI), which was 1 at baseline and represented 100% of patients received the biologic therapy. When the MD dropped to for example 0.50 in following years, 50% of patients remained on the same biologic therapy. When the MD was 0, all the patients discontinued the biologic therapy.

We further conducted a subgroup analysis limited to biologic-naïve subjects. The drug survival as to discontinuation due to loss of efficacy and adverse effects was analyzed separately. The Review Manager 5.3 (Copenhagen: The Nordic Cochrane Centre, The Cochrane Collaboration, 2014) was used for conducting meta-analysis.

## Electronic supplementary material

Supplement 1
