# Supplementary material for: Drug survival of biologics in treating psoriasis: a meta-analysis of real-world evidence
Source: Sci Rep. 2018 Oct 30;8:16068. doi: 10.1038/s41598-018-34293-y (PMC6207685; doi:10.1038/s41598-018-34293-y)
Supplement: Supplementary file 1 — Supplement 1 [file 41598_2018_34293_MOESM1_ESM.pdf]

Supplementary Material

Drug survival of biologics in treating psoriasis: a meta-analysis of real-world evidence

Pei-Tzu Lin, MS,[a,b] Shu-Hui Wang, MD, MS,[c,d] and Ching-Chi Chi, MD, MMS, DPhil[e,f]*

[a]Department of Pharmacy, Chang Gung Memorial Hospital, Chiayi, Chiayi, Taiwan

[b]Department of Nursing, Chang Gung University of Science and Technology, Chiayi, Taiwan

[c]Department of Dermatology, Far Eastern Memorial Hospital, New Taipei, Taiwan

[d]Graduate Institute of Applied Science and Engineering, College of Science and Engineering, Fu Jen Catholic University, New Taipei, Taiwan

[e]Department of Dermatology, Chang Gung Memorial Hospital, Linkou, Taoyuan, Taiwan

[f]College of Medicine, Chang Gung University, Taoyuan, Taiwan

***Correspondence:** C.-C. Chi. E-mail: chingchi@cgmh.org.tw

Figure legends for supplementary figures

Supplementary Table S1: Included studies of drug survival of biologics for treating psoriasis.

Supplementary Figure S1: Forest plots of drug survival of biologics for treating psoriasis in all subjects (a-d) with etanercept (a); with adalimumab (b); (c) with infliximab (c); with ustekinumab (d).

Supplementary Figure S2: Forest plots of drug survival of biologics for treating psoriasis in biologic-naïve subjects (a-d) with etanercept (a); with adalimumab (b); (c) with infliximab (c); with ustekinumab (d).

Supplementary Figure S3: Forest plots of drug survival for discontinue due to loss of efficacy (a-d) with etanercept (a); with adalimumab (b); (c) with infliximab (c); with ustekinumab (d).

Supplementary Figure S4: Forest plots of drug survival for discontinue due to adverse effects (a-d) with etanercept (a); with adalimumab (b); with infliximab (c); with ustekinumab (d).

Supplementary Table S1. Included studies of drug survival of biologics for treating psoriasis

References	Patient papulation	Drug survival rate								NOS score [a]	Note
		1 year		2 years		3 years		4 year			
	n	N	%	n	%	n	%	n	%		
**Etanercept**											
Arnold et al [9]	46	28	60	22	48	17	38	-	-	S****; C*; O**	
Bonafede et al[10]	1855	899	48.5	-	-	-	-	-	-	S****; C; O**	Biologic-naïve
Brunasso et al[11]	34	21	62.0	21	62.0	21	62.0	21	62.0	S****; C*; O**	Biologic-naïve
	13	4	30.0	2	14.0	2	14.0	2	14.0		Biologic-naïve
Chan et al[12]	1096	877	80.0	-	-	-	-	-	-	S****; C; O**	
Davila-Seijo et al[13]	672	388	82.9	258	49.2	168	38.0	129.0	20.5	S****; C*; O**	
Doshi et al[14]	1025	301	29.4	-	-	-	-	-	-	S****; C*; O**	
Esposito et al[15]	389	362	93.0	327	84.1	302	77.6	293	75.2	S****; C*; O**	
Gniadecki et al[16]	449	295	65.7	236	52.5	86	19.2	159	35.4	S****; C*; O**	
Gniadecki et al[17]	311	229	73.5	183	58.8	146	47.1	128	41.2	S****; C*; O**	
Inzinger et al[18]	501	355	70.8	305	60.8	281	56.0	-	-	S****; C*; O**	
Jacobi et al[19]	55	37	67.3	20	36.4	22	40.0	18	32.0	S****; C*; O**	
Kimball et al[20]	2510	1629	64.9	1329	52.9	1220	48.6	1076	42.9	S***; C; O**	
Di Lernia et al[21]	131	86	65.6							S****; C; O**	

References	Patient papulation	Drug survival rate								NOS score [a]	Note
		1 year		2 years		3 years		4 year			
		N	%	n	%	n	%	n	%		
Di Lernia et al [21]	116			43	37.1					S****; C; O**	
Marinas et al[22]	175	123	82.6	90	68.7	60	56.3	33	47.5	S****; C; O**	
Menter et al[23]	289	196	67.7	168	58.1	154	53.2	140	48.4		Biologic-naïve
	155	90	58.1	75	48.4	65	41.9	55	35.5	S****; C**; O**	2nd line therapy
	71	39	54.8	36	50.0	34	48.6	25	35.5		3rd line therapy
Menting et al[24]	118	100	85.0	94	79.6	91	77.0	76	64.0	S***; C; O**	Biologic-naïve
	55	47	86.0	39	71.4	35	62.9	23	42.0		Biologic-experienced
Mercadal et al[25]	34	-	-	21	62	-	-	-	-	S****; C*; O**	
Mitratza et al[26]	58	37	64.0	-	-	15	26.0	-	-	S****; C*; O**	Biologic-naïve
Pogácsás et al[27]	330	241	72.9	195	59.2	156	47.4			S****; C*; O**	
Richter et al[28]	55	28	50.9	13	23.6	5	9.1	4	7.3	S****; C*; O**	
Ross et al[29]	21	12	59.3	11	52.5	10	49.2	10	49.2	S****; C**; O**	
Shalom et al[30]	646	167	25.87	82	12.7	41	6.35			S****; C**; O**	
Van den Reek et al[31]	82	56	68.0	-	-	-	-	-	-	S****; C*; O**	
Van den Reek et al[32]	148	106	71.8	85	57.7	69	46.8	51	34.6	S****; C*; O**	Biologic-naïve
	45	38	84.6	31	68.0	28	62.8	25	55.8		Biologic-experienced
Ventayol et al[33]	34	-	-	21	62.0	-	-	-	-	S****; C; O**	Biologic-naïve

References	Patient papulation	Drug survival rate								NOS score [a]	Note
		1 year		2 years		3 years		4 year			
	n	N	%	n	%	n	%	n	%		
Vilarrasa et al [34]	248	176	70.9	130	52.6	97	39.1	76	30.5	S****; C**; O**	
Warren et al[35]	1098	769	70.0	560	51.0	439	40.0	-	-	S****; C*; O**	
Zweegers et al[36]	238	178	74.6	143	60.3	117	49.3	98	41.1	S****; C*; O**	
**Adalimumab**											
Arnold et al [9]	137	96	70	73	53	67	49	-	-	S****; C*; O**	
Bonafede et al[10]	1901	802	49.2	-	-	-	-	-	-	S****; C; O**	Biologic-naïve
Brunasso et al[11]	7	7	100.0	7	100.0	7	100.0	7	100.0	S****; C*; O**	Biologic-naïve
	8	6	74.0	6	74.0	-	-	-	-		Biologic-experienced
Davila-Seijo et al[13]	724	473	65.4	334	46.2	242	33.5	195	26.9	S****; C*; O**	
Doshi et al[14]	1084	438	40.4	-	-	-	-	-	-	S****; C*; O**	
Esposito et al[15]	114	99	86.5	80	70.1	67	58.4	56	49.5	S****; C*; O**	
Gniadecki et al[16]	774	524	67.7	481	62.1	414	53.5	379	49.0	S****; C*; O**	
Gniadecki et al[17]	427	289	67.7	214	50.0	163	38.2	151	35.3	S****; C*; O**	
Inzinger et al[18]	460	326	70.9	262	57.0	237	51.6	-	-	S****; C*; O**	
Jacobi et al[19]	37	22	59.5	14	37.8	20	55.2	16	43.2	S****; C*; O**	
Di Lernia et al [21]	78	51	65.4							S****; C; O**	
Di Lernia et al [21]	58			27	46.6					S****; C; O**	

References	Patient papulation	Drug survival rate								NOS score [a]	Note
		1 year		2 years		3 years		4 year			
		N	%	n	%	n	%	n	%		
Lopez-Ferrer et al[37]	49	38	78.5	38	78.5	38	78.5	32	65.9	S***; C*; O**	Biologic-naïve
	70	42	60.3	33	47.7	32	45.8	32	45.8		Biologic-experienced
Marinas et al[22]	134	82	80.4	53	64.8	28	55	19	50.8	S****; C; O**	
Menter et al[23]	402	318	79.2	263	65.5	263	65.5	188	46.8	S****; C**; O**	Biologic-naïve
	622	441	71.0	371	59.7	341	54.8	311	50.0		2nd line therapy
	197	130	66.1	108	54.8	95	48.4	79	40.3		3rd line therapy
Menting et al[24]	29	22	77.0	22	77.0	22	77.0	22	77.0	S***; C; O**	Biologic-naïve
	58	49	84.0	43	74.3	35	60.0	32	56.0		Biologic-experienced
Mercadal et al[25]	37			18	48					S****; C*; O**	
Mitratza et al[26]	30	16	51.9	-	-	7	22.2	-	-	S*****; C*; O**	Biologic-naïve
Pogácsás et al[27]	491	329	67.1	284	57.9	246	50			S****; C*; O**	
Richter et al[28]	106	68	64.1	52	49.1	34	32.1	24	22.6	S****; C*; O**	
Ross et al[29]	24	18	74.6	13	54.2	9	37.3	9	37.3	S****; C**; O**	
Shalom et al[30]	554	167	30.2	163	29.4	106	19.1			S****; C**; O**	
Spertino et al[38]	63	43	67.7	33	52.3	26	41.5	25	40.0	S****; C*; O**	
Suarez-Perez et al[39]	50	-	-	-	-	35	70.0	-	-	S****; C*; O**	
Umezawa et al[40]	59	47	79.7	-	-	-	-	-	-	S****; C; O**	

References	Patient papulation	Drug survival rate								NOS score [a]	Note
		1 year		2 years		3 years		4 year			
		N	%	n	%	n	%	n	%		
Van den Reek et al[41]	459	362	78.8	275	60.0	253	55.1	243	53.0	S***; C*; O**	
Van den Reek et al[42]	116	88	76.0	66	57.0	65	56.0	60	52.0	S***; C*; O**	
Van den Reek et al[31]	101	75	74.0	-	-	-	-	-	-	S****; C*; O**	
Van den Reek et al[43]	116	-	-	-	-	-	-	58	50.0	S****; C*; O**	
Ventayol et al[33]	37	-	-	18	48.0	-	-	-	-	S****; C; O**	Biologic-naïve
Vilarrasa et al[34]	231	159	69.0	123	53.2	97	41.9	81	34.9	S****; C**; O**	
Warren et al[35]	1879	1484	79.0	1259	67.0	1109	59.0			S****; C*; O**	
Zweegers et al[36]	186	141	75.8	112	60.3	102	54.8	89	47.9	S****; C*; O**	
**Infliximab**											
Arnold et al [9]	140	74	53	52	37	52	37	-	-	S****; C*; O**	
Brunasso et al[11]	33	6	18.0	5	14.0	5	14.0	5	14.0	S****; C*; O**	Biologic-naïve
	2	2	100.0	2	100.0	2	100.0	2	100.0		Biologic-experienced
Davila-Seijo et al[13]	188	123	65.4	87	46.2	65	34.6	54	28.9	S****; C*; O**	
Doshi et al[14]	318	157	49.4	-	-	-	-	-	-	S****; C*; O**	
Esposito et al[15]	147	117	79.4	89	60.8	70	47.7	57	38.8	S****; C*; O**	
Gniadecki et al[16]	253	180	71.2	146	57.6	130	51.5	111	43.9	S****; C*; O**	
Gniadecki et al[17]	144	123	85.3	110	76.5	102	70.6	97	67.7	S****; C*; O**	

References	Patient papulation	Drug survival rate								NOS score [a]	Note
		1 year		2 years		3 years		4 year			
	n	N	%	n	%	n	%	n	%		
Inzinger et al[18]	58	34	58.0	17	30.1	13	22.6	-	-	S****; C*; O**	
Jacobi et al[19]	13	7	7.0	3	3.0	-	-	-	-	S****; C*; O**	
Di Lernia et al[21]	98	70	64.6							S****; C; O**	
Di Lernia et al[21]	89			41	37.1					S****; C; O**	
Marinas et al[22]	160	40	83.8	30	66.4	25	55.4	19	48.5	S****; C; O**	
Menter et al[23]	63	53	83.9	49	77.4	43	67.7	34	53.2	S****; C**; O**	Biologic-naïve
	93	63	67.7	51	54.8	24	25.8	15	16.1		2nd line therapy
	103	71	69.4	58	56.5	40	38.7	37	35.5		3rd line therapy
Menting et al[24]	19	14	75.0	14	75.0	14	75.0	14	75.0	S***; C; O**	Biologic-naïve
	21	14	68.0	13	60.0	9	42.9	9	43.0		Biologic-experienced
Mitratza et al[26]	21	11	54.4	-	-	9	42.9	-	-	S*****; C*; O**	Biologic-naïve
Pogácsás et al[27]	266	182	68.4	144	54.0	91	34.2	-	-	S****; C*; O**	
Ross et al[29]	14	10	69.5	10	69.5	8	59.3	6	41.5	S****; C**; O**	
Shalom et al[30]	152	46	30.2	29	19.1	22	14.3			S****; C**; O**	
Umezawa et al[40]	38	28	73.3	-	-	-	-	-	-	S****; C; O**	
Vilarrasa et al[34]	84	58	68.8	46	54.2	38	45.5	36	42.4	S****; C**; O**	
Warren et al[35]	96	62	65.0	48	50.0	34	35.0	-	-	S****; C*; O**	

References	Patient papulation	Drug survival rate								NOS score [a]	Note
		1 year		2 years		3 years		4 year			
		N	%	n	%	n	%	n	%		
**Ustekinumab**											
Arnold et al [9]	59	53	90.0	49	83.0	49	83.0	-	-	S****; C*; O**	
Davila-Seijo et al[13]	491	397	80.8	312	63.5	246	50.0	208	42.3	S****; C*; O**	
Doshi et al[14]	280	121	43.2	-	-	-	-	-	-	S****; C*; O**	
García-Martínez et al[44]	35	32	90.0	25	71.0	24	69.0	-	-	S***; C; O**	
Gniadecki et al[16]	391	308	78.8	278	71.2	271	69.2	263	67.2	S****; C*; O**	
Jacobi et al[19]	11	11	100.0	6	54.6	8	76.0	-	-	S****; C*; O**	
Marinas et al[22]	176	122	91.4	85	85.6	47	80.0	18	75.5	S****; C; O**	
Menter et al[23]	301	282	93.6	272	90.3	262	87.1	262	87.1	S****; C**; O**	Biologic-naïve
	566	493	87.1	456	80.7	438	77.4	438	77.4		2nd line therapy
	551	462	83.9	427	77.4	391	71.0	373	67.7		3rd line therapy
Menting et al[24]	1	-	-	-	-	-	-	-	-	S***; C; O**	Biologic-naïve
	21	18	84.0	14	65.7	12	57.0	-	-		Biologic-experienced
Mercadal et al[25]	41	-	-	33	81.0	-	-	-	-	S****; C*; O**	
Mitratza et al[26]	25	21	82.6	-	-	12	47.8	-	-	S*****; C*; O**	Biologic-naïve
Pogácsás et al[27]	487	410	84.2	352	72.3	320	65.8			S****; C*; O**	

References	Patient papulation	Drug survival rate								NOS score [a]	Note
		1 year		2 years		3 years		4 year			
		N	%	n	%	n	%	n	%		
Richter et al[28]	48	34	70.8	18	37.5	7	14.6	2	4.2	S****; C*; O**	
Ross et al[29]	25	23	93.2	23	93.2	-	-	-	-	S****; C**; O**	
Shalom et al[30]	224	171	76.2	149	66.7	139	61.9			S****; C**; O**	
Suarez-Perez et al[45]	44	-	-	-	-	37	84.0	-	-	S***; C*; O**	
Umezawa et al[40]	30	29	96.7	-	-	-	-	-	-	S****; C; O**	
Van den Reek et al[31]	66	56	85.0	-	-	-	-	-	-	S****; C*; O**	
Ventayol et al[33]	41	-	-	33	81.0	-	-	-	-	S****; C; O**	Biologic-naïve
Vilarrasa et al[34]	140	108	77.2	96	68.5	87	61.8	81	57.8	S****; C**; O**	
Warren et al[35]	450	401	89.0	369	82.0	338	75.0	-	-	S****; C*; O**	
Zweegers et al[36]	102	86	84.0	75	73.6	73	71.2	73	71.2	S****; C*; O**	

NOS Newcastle–Ottawa scale

[a] :Scale domains: S selection of study groups, C comparability, O outcome assessment

Supplementary Figure S1(a): Forest plots of drug survival of biologics for treating psoriasis in all subjects with etanercept.

Study or Subgroup	Risk Difference	SE	Weight	Mean difference IV, Random, 95% CI
**1.1.1 Year 1**				
Arnold 2016	0.6	0.0106	3.8%	0.60 [0.58, 0.62]
Bonafeda 2013	0.485	0.0003	3.9%	0.48 [0.48, 0.49]
Brunasso 2012	0.54	0.0106	3.8%	0.54 [0.52, 0.56]
Chan 2010	0.8	0.0004	3.9%	0.80 [0.80, 0.80]
Davila-Seijo 2016	0.829	0.0006	3.9%	0.83 [0.83, 0.83]
Di Lernia 2012	0.656	0.0036	3.8%	0.66 [0.65, 0.66]
Doshi 2016	0.294	0.0004	3.9%	0.29 [0.29, 0.29]
Esposito 2013	0.93	0.0007	3.9%	0.93 [0.93, 0.93]
Gniadecki 2011	0.735	0.0014	3.9%	0.73 [0.73, 0.74]
Gniadecki 2015	0.657	0.0011	3.9%	0.66 [0.65, 0.66]
Inzinger 2016	0.708	0.0009	3.9%	0.71 [0.71, 0.71]
Jacobi 2016	0.673	0.0085	3.8%	0.67 [0.66, 0.69]
Kimball 2015	0.649	0.0002	3.9%	0.65 [0.65, 0.65]
Marinas 2016	0.826	0.0022	3.9%	0.83 [0.82, 0.83]
Menter 2016	0.63	0.0009	3.9%	0.63 [0.63, 0.63]
Menting 2014	0.853	0.002	3.9%	0.85 [0.85, 0.86]
Mitratza 2017	0.64	0.0083	3.8%	0.64 [0.62, 0.66]
Pogácsás 2017	0.729	0.0013	3.9%	0.73 [0.73, 0.73]
Richter 2017	0.509	0.0091	3.8%	0.51 [0.49, 0.53]
Ross 2016	0.593	0.0234	3.8%	0.59 [0.55, 0.64]
Shalom 2016	0.259	0.0007	3.9%	0.26 [0.26, 0.26]
Van den Reek 2014a	0.77	0.0022	3.9%	0.77 [0.77, 0.77]
Van den Reek 2014b	0.68	0.0057	3.8%	0.68 [0.67, 0.69]
Vilarrasa 2016	0.709	0.0018	3.9%	0.71 [0.71, 0.71]
Warren 2015	0.7	0.0004	3.9%	0.70 [0.70, 0.70]
Zweegers 2016	0.746	0.0018	3.9%	0.75 [0.74, 0.75]
**Subtotal (95% CI)**			**100.0%**	**0.66 [0.59, 0.73]**
Heterogeneity: $Tau^2 = 0.03$; $Chi^2 = 1772246.76$, df = 25 (P < 0.00001); $I^2 = 100\%$				
Test for overall effect: Z = 18.69 (P < 0.00001)				
**1.1.2 Year 2**				
Arnold 2016	0.48	0.0109	3.8%	0.48 [0.46, 0.50]
Brunasso 2012	0.48	0.0106	3.8%	0.48 [0.46, 0.50]
Chan 2010	0.8	0.0004	3.9%	0.80 [0.80, 0.80]
Davila-Seijo 2016	0.492	0.0007	3.9%	0.49 [0.49, 0.49]
Di Lernia 2012	0.371	0.0042	3.9%	0.37 [0.36, 0.38]
Doshi 2016	0.294	0.0004	3.9%	0.29 [0.29, 0.29]
Esposito 2013	0.841	0.0009	3.9%	0.84 [0.84, 0.84]
Gniadecki 2011	0.588	0.0016	3.9%	0.59 [0.58, 0.59]
Gniadecki 2015	0.525	0.0011	3.9%	0.53 [0.52, 0.53]
Inzinger 2016	0.608	0.001	3.9%	0.61 [0.61, 0.61]
Jacobi 2016	0.364	0.0087	3.8%	0.36 [0.35, 0.38]
Kimball 2015	0.529	0.0002	3.9%	0.53 [0.53, 0.53]
Marinas 2016	0.687	0.0026	3.9%	0.69 [0.68, 0.69]
Menter 2016	0.54	0.001	3.9%	0.54 [0.54, 0.54]
Menting 2014	0.77	0.0024	3.9%	0.77 [0.77, 0.77]
Mercadal 2016	0.62	0.0143	3.8%	0.62 [0.59, 0.65]
Pogácsás 2017	0.592	0.0015	3.9%	0.59 [0.59, 0.59]
Richter 2017	0.236	0.0077	3.8%	0.24 [0.22, 0.25]
Ross 2016	0.525	0.0238	3.8%	0.53 [0.48, 0.57]
Shalom 2016	0.127	0.0005	3.9%	0.13 [0.13, 0.13]
Van den Reek 2014a	0.594	0.0025	3.9%	0.59 [0.59, 0.60]
Van den Reek 2014b	0.594	0.0107	3.8%	0.59 [0.57, 0.61]
Ventayol 2014	0.62	0.0143	3.8%	0.62 [0.59, 0.65]
Vilarrasa 2016	0.526	0.002	3.9%	0.53 [0.52, 0.53]
Warren 2015	0.51	0.0005	3.9%	0.51 [0.51, 0.51]
Zweegers 2016	0.603	0.0021	3.9%	0.60 [0.60, 0.61]
**Subtotal (95% CI)**			**100.0%**	**0.54 [0.46, 0.61]**
Heterogeneity: $Tau^2 = 0.04$; $Chi^2 = 1587222.59$, df = 25 (P < 0.00001); $I^2 = 100\%$				
Test for overall effect: Z = 13.63 (P < 0.00001)				
**1.1.3 Year 3**				
Arnold 2016	0.38	0.0106	4.8%	0.38 [0.36, 0.40]
Brunasso 2012	0.48	0.0106	4.8%	0.48 [0.46, 0.50]
Davila-Seijo 2016	0.38	0.0007	4.8%	0.38 [0.38, 0.38]
Esposito 2013	0.776	0.0011	4.8%	0.78 [0.77, 0.78]
Gniadecki 2011	0.471	0.0016	4.8%	0.47 [0.47, 0.47]
Gniadecki 2015	0.192	0.0009	4.8%	0.19 [0.19, 0.19]
Inzinger 2016	0.56	0.001	4.8%	0.56 [0.56, 0.56]
Jacobi 2016	0.4	0.0089	4.8%	0.40 [0.38, 0.42]
Kimball 2015	0.486	0.0002	4.8%	0.49 [0.49, 0.49]
Marinas 2016	0.563	0.0028	4.8%	0.56 [0.56, 0.57]
Menter 2016	0.492	0.001	4.8%	0.49 [0.49, 0.49]
Menting 2014	0.725	0.0026	4.8%	0.72 [0.72, 0.73]
Mitratza 2017	0.26	0.0076	4.8%	0.26 [0.25, 0.27]
Pogácsás 2017	0.474	0.0015	4.8%	0.47 [0.47, 0.48]
Richter 2017	0.091	0.0052	4.8%	0.09 [0.08, 0.10]
Ross 2016	0.492	0.0238	4.7%	0.49 [0.45, 0.54]
Shalom 2016	0.064	0.0004	4.8%	0.06 [0.06, 0.06]
Van den Reek 2014a	0.506	0.0026	4.8%	0.51 [0.50, 0.51]
Vilarrasa 2016	0.391	0.002	4.8%	0.39 [0.39, 0.39]
Warren 2015	0.4	0.0004	4.8%	0.40 [0.40, 0.40]
Zweegers 2016	0.493	0.0021	4.8%	0.49 [0.49, 0.50]
**Subtotal (95% CI)**			**100.0%**	**0.43 [0.35, 0.52]**
Heterogeneity: $Tau^2 = 0.04$; $Chi^2 = 1121858.13$, df = 20 (P < 0.00001); $I^2 = 100\%$				
Test for overall effect: Z = 10.19 (P < 0.00001)				
**1.1.4 Year 4**				
Brunasso 2012	0.48	0.0106	6.6%	0.48 [0.46, 0.50]
Davila-Seijo 2016	0.205	0.0006	6.7%	0.20 [0.20, 0.21]
Esposito 2013	0.752	0.0011	6.7%	0.75 [0.75, 0.75]
Gniadecki 2011	0.412	0.0016	6.7%	0.41 [0.41, 0.42]
Gniadecki 2015	0.354	0.0011	6.7%	0.35 [0.35, 0.36]
Jacobi 2016	0.32	0.0085	6.7%	0.32 [0.30, 0.34]
Kimball 2015	0.429	0.0002	6.7%	0.43 [0.43, 0.43]
Marinas 2016	0.475	0.0029	6.7%	0.47 [0.47, 0.48]
Menter 2016	0.427	0.001	6.7%	0.43 [0.43, 0.43]
Menting 2014	0.57	0.0029	6.7%	0.57 [0.56, 0.58]
Richter 2017	0.073	0.0047	6.7%	0.07 [0.06, 0.08]
Ross 2016	0.492	0.0238	6.5%	0.49 [0.45, 0.54]
Van den Reek 2014a	0.41	0.0025	6.7%	0.41 [0.41, 0.41]
Vilarrasa 2016	0.305	0.0019	6.7%	0.30 [0.30, 0.31]
Zweegers 2016	0.411	0.0021	6.7%	0.41 [0.41, 0.42]
**Subtotal (95% CI)**			**100.0%**	**0.41 [0.34, 0.48]**
Heterogeneity: $Tau^2 = 0.02$; $Chi^2 = 236584.28$, df = 14 (P < 0.00001); $I^2 = 100\%$				
Test for overall effect: Z = 11.14 (P < 0.00001)				

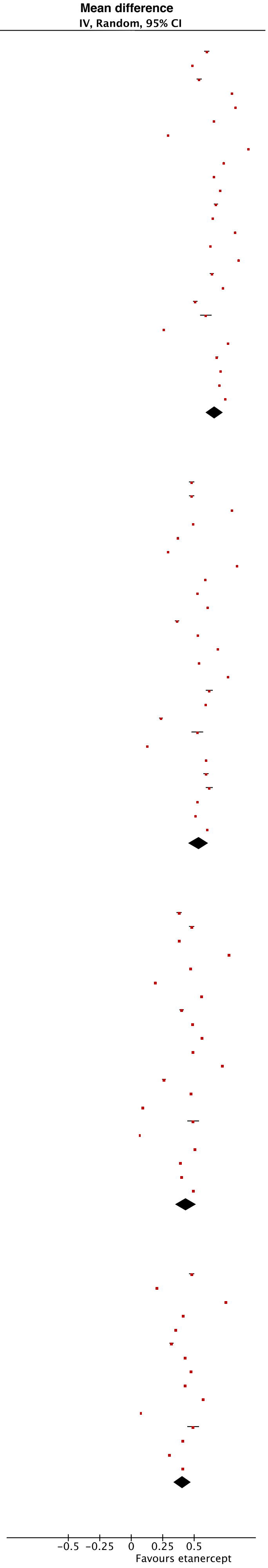

Supplementary Figure S1(b): Forest plots of drug survival of biologics for treating psoriasis in all subjects with adalimumab.

Study or Subgroup	Risk Difference	SE	Weight	Mean difference IV, Random, 95% CI
**1.2.1 Year 1**				
Arnold 2016	0.7	0.0033	3.6%	0.70 [0.69, 0.71]
Bonafeda 2013	0.492	0.0003	3.6%	0.49 [0.49, 0.49]
Brunasso 2012	0.86	0.0231	3.5%	0.86 [0.81, 0.91]
Davila–Seijo 2016	0.654	0.0007	3.6%	0.65 [0.65, 0.66]
Di Lernia 2012	0.654	0.0061	3.6%	0.65 [0.64, 0.67]
Doshi 2016	0.404	0.0005	3.6%	0.40 [0.40, 0.40]
Esposito 2013	0.865	0.003	3.6%	0.86 [0.86, 0.87]
Gniadecki 2011	0.677	0.0011	3.6%	0.68 [0.67, 0.68]
Gniadecki 2015	0.677	0.0006	3.6%	0.68 [0.68, 0.68]
Inzinger 2016	0.709	0.001	3.6%	0.71 [0.71, 0.71]
Jacobi 2016	0.595	0.0133	3.6%	0.59 [0.57, 0.62]
Lopez–Ferrer 2013	0.678	0.0039	3.6%	0.68 [0.67, 0.69]
Marinas 2016	0.804	0.003	3.6%	0.80 [0.80, 0.81]
Menter 2016	0.729	0.0004	3.6%	0.73 [0.73, 0.73]
Menting 2014	0.817	0.0044	3.6%	0.82 [0.81, 0.83]
Mitratza 2017	0.519	0.0167	3.5%	0.52 [0.49, 0.55]
Pogácsás 2017	0.671	0.001	3.6%	0.67 [0.67, 0.67]
Richter 2017	0.642	0.0045	3.6%	0.64 [0.63, 0.65]
Ross 2016	0.746	0.0181	3.5%	0.75 [0.71, 0.78]
Shalom 2016	0.302	0.0008	3.6%	0.30 [0.30, 0.30]
Spertino 2014	0.677	0.0074	3.6%	0.68 [0.66, 0.69]
Umezawa 2013	0.797	0.0068	3.6%	0.80 [0.78, 0.81]
Van den Reek 2014b	0.74	0.0043	3.6%	0.74 [0.73, 0.75]
Van den Reek 2015	0.76	0.0037	3.6%	0.76 [0.75, 0.77]
Van den Reek 2016	0.788	0.0009	3.6%	0.79 [0.79, 0.79]
Vilarrasa 2016	0.69	0.002	3.6%	0.69 [0.69, 0.69]
Warren 2015	0.79	0.0002	3.6%	0.79 [0.79, 0.79]
Zweegers 2016	0.758	0.0023	3.6%	0.76 [0.75, 0.76]
**Subtotal (95% CI)**			**100.0%**	**0.69 [0.62, 0.75]**
Heterogeneity: $Tau^2 = 0.03$; $Chi^2 = 1258271.88$, df = 27 (P < 0.00001); $I^2 = 100\%$				
Test for overall effect: Z = 21.36 (P < 0.00001)				
**1.2.2 Year 2**				
Arnold 2016	0.53	0.0036	4.0%	0.53 [0.52, 0.54]
Brunasso 2012	0.86	0.0231	3.8%	0.86 [0.81, 0.91]
Davila–Seijo 2016	0.462	0.0007	4.0%	0.46 [0.46, 0.46]
Di Lernia 2012	0.466	0.0086	4.0%	0.47 [0.45, 0.48]
Esposito 2013	0.701	0.004	4.0%	0.70 [0.69, 0.71]
Gniadecki 2011	0.5	0.0012	4.0%	0.50 [0.50, 0.50]
Gniadecki 2015	0.621	0.0006	4.0%	0.62 [0.62, 0.62]
Inzinger 2016	0.57	0.0011	4.0%	0.57 [0.57, 0.57]
Jacobi 2016	0.378	0.0131	4.0%	0.38 [0.35, 0.40]
Lopez–Ferrer 2013	0.604	0.0041	4.0%	0.60 [0.60, 0.61]
Marinas 2016	0.648	0.0036	4.0%	0.65 [0.64, 0.66]
Menter 2016	0.608	0.0004	4.0%	0.61 [0.61, 0.61]
Menting 2014	0.752	0.005	4.0%	0.75 [0.74, 0.76]
Mercadal 2016	0.48	0.0135	4.0%	0.48 [0.45, 0.51]
Pogácsás 2017	0.579	0.001	4.0%	0.58 [0.58, 0.58]
Richter 2017	0.491	0.0047	4.0%	0.49 [0.48, 0.50]
Ross 2016	0.542	0.0208	3.9%	0.54 [0.50, 0.58]
Shalom 2016	0.294	0.0008	4.0%	0.29 [0.29, 0.30]
Spertino 2014	0.523	0.0079	4.0%	0.52 [0.51, 0.54]
Van den Reek 2015	0.57	0.0043	4.0%	0.57 [0.56, 0.58]
Van den Reek 2016	0.6	0.0011	4.0%	0.60 [0.60, 0.60]
Ventayol 2014	0.48	0.0135	4.0%	0.48 [0.45, 0.51]
Vilarrasa 2016	0.532	0.0022	4.0%	0.53 [0.53, 0.54]
Warren 2015	0.67	0.0003	4.0%	0.67 [0.67, 0.67]
Zweegers 2016	0.603	0.0026	4.0%	0.60 [0.60, 0.61]
**Subtotal (95% CI)**			**100.0%**	**0.56 [0.52, 0.61]**
Heterogeneity: $Tau^2 = 0.01$; $Chi^2 = 253949.04$, df = 24 (P < 0.00001); $I^2 = 100\%$				
Test for overall effect: Z = 25.78 (P < 0.00001)				
**1.2.3 Year 3**				
Arnold 2016	0.49	0.0036	4.2%	0.49 [0.48, 0.50]
Brunasso 2012	0.86	0.0231	4.1%	0.86 [0.81, 0.91]
Davila–Seijo 2016	0.335	0.0007	4.2%	0.34 [0.33, 0.34]
Esposito 2013	0.584	0.0043	4.2%	0.58 [0.58, 0.59]
Gniadecki 2011	0.382	0.0011	4.2%	0.38 [0.38, 0.38]
Gniadecki 2015	0.535	0.0006	4.2%	0.54 [0.53, 0.54]
Inzinger 2016	0.516	0.0011	4.2%	0.52 [0.51, 0.52]
Jacobi 2016	0.552	0.0134	4.1%	0.55 [0.53, 0.58]
Lopez–Ferrer 2013	0.592	0.0041	4.2%	0.59 [0.58, 0.60]
Marinas 2016	0.55	0.0037	4.2%	0.55 [0.54, 0.56]
Menter 2016	0.573	0.0004	4.2%	0.57 [0.57, 0.57]
Menting 2014	0.657	0.0055	4.2%	0.66 [0.65, 0.67]
Mitratza 2017	0.222	0.0139	4.1%	0.22 [0.19, 0.25]
Pogácsás 2017	0.5	0.001	4.2%	0.50 [0.50, 0.50]
Richter 2017	0.321	0.0044	4.2%	0.32 [0.31, 0.33]
Ross 2016	0.373	0.0202	4.1%	0.37 [0.33, 0.41]
Shalom 2016	0.191	0.0007	4.2%	0.19 [0.19, 0.19]
Spertino 2014	0.415	0.0078	4.2%	0.41 [0.40, 0.43]
Suarez–Perez 2014	0.7	0.0092	4.2%	0.70 [0.68, 0.72]
Van den Reek 2015	0.56	0.0043	4.2%	0.56 [0.55, 0.57]
Van den Reek 2016	0.551	0.0011	4.2%	0.55 [0.55, 0.55]
Ventayol 2014	0	0		Not estimable
Vilarrasa 2016	0.419	0.0021	4.2%	0.42 [0.41, 0.42]
Warren 2015	0.59	0.0003	4.2%	0.59 [0.59, 0.59]
Zweegers 2016	0.548	0.0027	4.2%	0.55 [0.54, 0.55]
**Subtotal (95% CI)**			**100.0%**	**0.50 [0.45, 0.55]**
Heterogeneity: $Tau^2 = 0.02$; $Chi^2 = 387432.92$, df = 23 (P < 0.00001); $I^2 = 100\%$				
Test for overall effect: Z = 18.50 (P < 0.00001)				
**1.2.4 Year 4**				
Brunasso 2012	0.86	0.0231	5.3%	0.86 [0.81, 0.91]
Davila–Seijo 2016	0.269	0.0006	5.6%	0.27 [0.27, 0.27]
Esposito 2013	0.495	0.0044	5.6%	0.49 [0.49, 0.50]
Gniadecki 2011	0.353	0.0011	5.6%	0.35 [0.35, 0.36]
Gniadecki 2015	0.49	0.0006	5.6%	0.49 [0.49, 0.49]
Jacobi 2016	0.432	0.0134	5.5%	0.43 [0.41, 0.46]
Lopez–Ferrer 2013	0.541	0.0042	5.6%	0.54 [0.53, 0.55]
Marinas 2016	0.508	0.0037	5.6%	0.51 [0.50, 0.52]
Menter 2016	0.474	0.0004	5.6%	0.47 [0.47, 0.47]
Menting 2014	0.63	0.0055	5.6%	0.63 [0.62, 0.64]
Richter 2017	0.226	0.0039	5.6%	0.23 [0.22, 0.23]
Ross 2016	0.373	0.0202	5.4%	0.37 [0.33, 0.41]
Spertino 2014	0.4	0.0078	5.6%	0.40 [0.38, 0.42]
Van den Reek 2014c	0.5	0.0043	5.6%	0.50 [0.49, 0.51]
Van den Reek 2015	0.52	0.0043	5.6%	0.52 [0.51, 0.53]
Van den Reek 2016	0.53	0.0011	5.6%	0.53 [0.53, 0.53]
Vilarrasa 2016	0.349	0.0021	5.6%	0.35 [0.34, 0.35]
Zweegers 2016	0.479	0.0027	5.6%	0.48 [0.47, 0.48]
**Subtotal (95% CI)**			**100.0%**	**0.47 [0.42, 0.52]**
Heterogeneity: $Tau^2 = 0.01$; $Chi^2 = 115550.37$, df = 17 (P < 0.00001); $I^2 = 100\%$				
Test for overall effect: Z = 18.67 (P < 0.00001)				

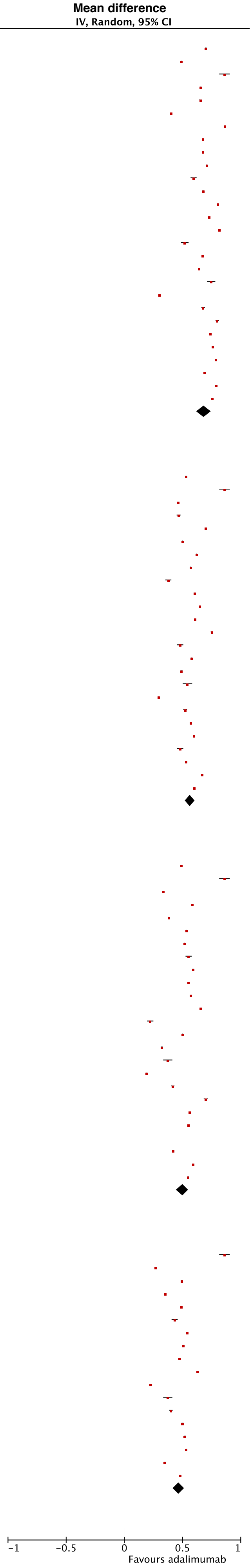

Supplementary Figure S1(c): Forest plots of drug survival of biologics for treating psoriasis in all subjects with infliximab.

Study or Subgroup	Risk Difference	SE	Weight	Mean difference IV, Random, 95% CI
**1.3.1 Year 1**				
Arnold 2016	0.53	0.0036	5.0%	0.53 [0.52, 0.54]
Brunasso 2012	0.2	0.0114	5.0%	0.20 [0.18, 0.22]
Davila-Seijo 2016	0.654	0.0025	5.0%	0.65 [0.65, 0.66]
Di Lernia 2012	0.656	0.0048	5.0%	0.66 [0.65, 0.67]
Doshi 2016	0.494	0.0016	5.0%	0.49 [0.49, 0.50]
Esposito 2013	0.794	0.0028	5.0%	0.79 [0.79, 0.80]
Gniadecki 2011	0.853	0.0025	5.0%	0.85 [0.85, 0.86]
Gniadecki 2015	0.712	0.0018	5.0%	0.71 [0.71, 0.72]
Inzinger 2016	0.58	0.0085	5.0%	0.58 [0.56, 0.60]
Jacobi 2016	0.07	0.0196	4.9%	0.07 [0.03, 0.11]
Marinas 2016	0.838	0.0023	5.0%	0.84 [0.83, 0.84]
Menter 2016	0.723	0.0017	5.0%	0.72 [0.72, 0.73]
Menting 2014	0.713	0.0113	5.0%	0.71 [0.69, 0.74]
Mitratza 2017	0.524	0.0238	4.9%	0.52 [0.48, 0.57]
Pogácsás 2017	0.684	0.0017	5.0%	0.68 [0.68, 0.69]
Ross 2016	0.695	0.0329	4.8%	0.69 [0.63, 0.76]
Shalom 2016	0.302	0.003	5.0%	0.30 [0.30, 0.31]
Umezawa 2013	0.733	0.0116	5.0%	0.73 [0.71, 0.76]
Vilarrasa 2016	0.688	0.0055	5.0%	0.69 [0.68, 0.70]
Warren 2015	0.65	0.005	5.0%	0.65 [0.64, 0.66]
**Subtotal (95% CI)**			**100.0%**	**0.61 [0.54, 0.67]**

Heterogeneity: $Tau^2 = 0.02$; $Chi^2 = 45688.87$, df = 19 ($P < 0.00001$); $I^2 = 100\%$
Test for overall effect: Z = 18.36 ($P < 0.00001$)

Study or Subgroup	Risk Difference	SE	Weight	Mean difference IV, Random, 95% CI
**1.3.2 Year 2**				
Arnold 2016	0.37	0.0034	5.9%	0.37 [0.36, 0.38]
Brunasso 2012	0.16	0.0105	5.9%	0.16 [0.14, 0.18]
Davila-Seijo 2016	0.462	0.0027	5.9%	0.46 [0.46, 0.47]
Di Lernia 2012	0.371	0.0054	5.9%	0.37 [0.36, 0.38]
Esposito 2013	0.608	0.0033	5.9%	0.61 [0.60, 0.61]
Gniadecki 2011	0.765	0.0029	5.9%	0.77 [0.76, 0.77]
Gniadecki 2015	0.576	0.002	5.9%	0.58 [0.57, 0.58]
Inzinger 2016	0.301	0.0079	5.9%	0.30 [0.29, 0.32]
Jacobi 2016	0.03	0.0131	5.9%	0.03 [0.00, 0.06]
Marinas 2016	0.664	0.003	5.9%	0.66 [0.66, 0.67]
Menter 2016	0.61	0.0019	5.9%	0.61 [0.61, 0.61]
Menting 2014	0.671	0.0117	5.9%	0.67 [0.65, 0.69]
Pogácsás 2017	0.579	0.001	5.9%	0.58 [0.58, 0.58]
Ross 2016	0.695	0.0329	5.6%	0.69 [0.63, 0.76]
Shalom 2016	0.191	0.0026	5.9%	0.19 [0.19, 0.20]
Vilarrasa 2016	0.542	0.0059	5.9%	0.54 [0.53, 0.55]
Warren 2015	0.5	0.0052	5.9%	0.50 [0.49, 0.51]
**Subtotal (95% CI)**			**100.0%**	**0.48 [0.41, 0.54]**

Heterogeneity: $Tau^2 = 0.02$; $Chi^2 = 37451.64$, df = 16 ($P < 0.00001$); $I^2 = 100\%$
Test for overall effect: Z = 13.72 ($P < 0.00001$)

Study or Subgroup	Risk Difference	SE	Weight	Mean difference IV, Random, 95% CI
**1.3.3 Year 3**				
Arnold 2016	0.37	0.0034	6.3%	0.37 [0.36, 0.38]
Brunasso 2012	0.16	0.0105	6.3%	0.16 [0.14, 0.18]
Davila-Seijo 2016	0.346	0.0025	6.3%	0.35 [0.34, 0.35]
Esposito 2013	0.477	0.0034	6.3%	0.48 [0.47, 0.48]
Gniadecki 2011	0.706	0.0032	6.3%	0.71 [0.70, 0.71]
Gniadecki 2015	0.515	0.002	6.3%	0.52 [0.51, 0.52]
Inzinger 2016	0.226	0.0072	6.3%	0.23 [0.21, 0.24]
Marinas 2016	0.554	0.0031	6.3%	0.55 [0.55, 0.56]
Menter 2016	0.411	0.0019	6.3%	0.41 [0.41, 0.41]
Menting 2014	0.581	0.0123	6.2%	0.58 [0.56, 0.61]
Mitratza 2017	0.222	0.0139	6.2%	0.22 [0.19, 0.25]
Pogácsás 2017	0.5	0.001	6.3%	0.50 [0.50, 0.50]
Ross 2016	0.593	0.0351	5.9%	0.59 [0.52, 0.66]
Shalom 2016	0.143	0.0023	6.3%	0.14 [0.14, 0.15]
Vilarrasa 2016	0.455	0.0059	6.3%	0.46 [0.44, 0.47]
Warren 2015	0.35	0.005	6.3%	0.35 [0.34, 0.36]
**Subtotal (95% CI)**			**100.0%**	**0.41 [0.35, 0.48]**

Heterogeneity: $Tau^2 = 0.02$; $Chi^2 = 34196.86$, df = 15 ($P < 0.00001$); $I^2 = 100\%$
Test for overall effect: Z = 12.06 ($P < 0.00001$)

Study or Subgroup	Risk Difference	SE	Weight	Mean difference IV, Random, 95% CI
**1.3.4 Year 4**				
Brunasso 2012	0.16	0.0105	10.0%	0.16 [0.14, 0.18]
Davila-Seijo 2016	0.289	0.0024	10.1%	0.29 [0.28, 0.29]
Esposito 2013	0.388	0.0033	10.1%	0.39 [0.38, 0.39]
Gniadecki 2011	0.677	0.0032	10.1%	0.68 [0.67, 0.68]
Gniadecki 2015	0.439	0.002	10.1%	0.44 [0.44, 0.44]
Marinas 2016	0.485	0.0031	10.1%	0.48 [0.48, 0.49]
Menter 2016	0.329	0.0018	10.1%	0.33 [0.33, 0.33]
Menting 2014	0.582	0.0123	10.0%	0.58 [0.56, 0.61]
Ross 2016	0.415	0.0352	9.3%	0.41 [0.35, 0.48]
Vilarrasa 2016	0.424	0.0059	10.1%	0.42 [0.41, 0.44]
**Subtotal (95% CI)**			**100.0%**	**0.42 [0.34, 0.50]**

Heterogeneity: $Tau^2 = 0.02$; $Chi^2 = 13082.51$, df = 9 ($P < 0.00001$); $I^2 = 100\%$
Test for overall effect: Z = 10.74 ($P < 0.00001$)

Mean difference IV, Random, 95% CI

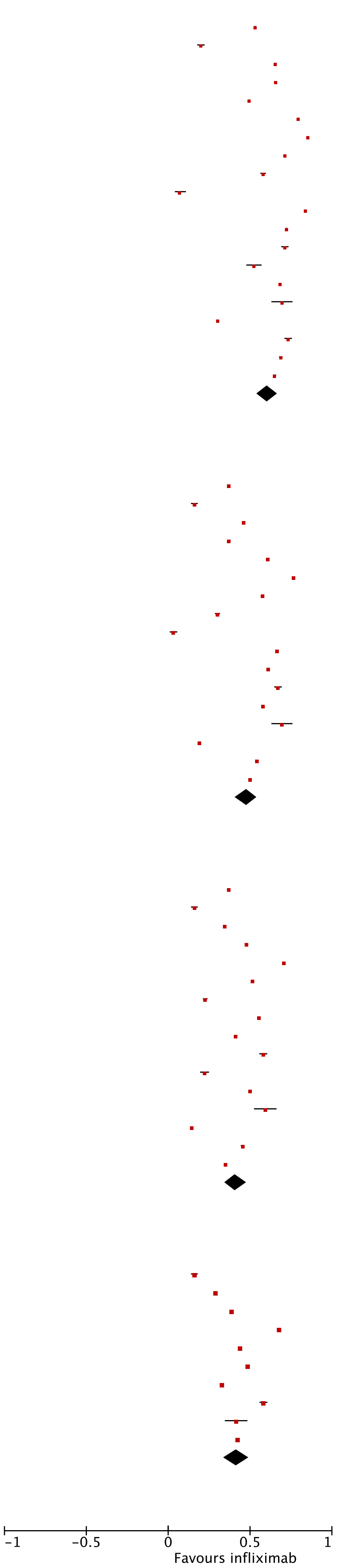

Test for subgroup differences: $Chi^2 = 20.71$, df = 3 ($P = 0.0001$), $I^2 = 85.5\%$

Supplementary Figure S1(d): Forest plots of drug survival of biologics for treating psoriasis in all subjects with ustekinumab.

Study or Subgroup	Risk Difference	SE	Weight	Mean difference IV, Random, 95% CI
**1.5.1 Year 1**				
Arnold 2016	0.9	0.0051	5.6%	0.90 [0.89, 0.91]
Davila-Seijo 2016	0.808	0.0008	5.6%	0.81 [0.81, 0.81]
Doshi 2016	0.432	0.0018	5.6%	0.43 [0.43, 0.44]
García-Martínez 2015	0.9	0.0086	5.5%	0.90 [0.88, 0.92]
Gniadecki 2015	0.788	0.001	5.6%	0.79 [0.79, 0.79]
Jacobi 2016	1	0		Not estimable
Marinas 2016	0.914	0.0016	5.6%	0.91 [0.91, 0.92]
Menter 2016	0.872	0.0002	5.6%	0.87 [0.87, 0.87]
Menting 2014	0.802	0.0181	5.3%	0.80 [0.77, 0.84]
Mitratza 2017	0.826	0.0152	5.4%	0.83 [0.80, 0.86]
Pogácsás 2017	0.842	0.0007	5.6%	0.84 [0.84, 0.84]
Richter 2017	0.708	0.0095	5.5%	0.71 [0.69, 0.73]
Ross 2016	0.932	0.0101	5.5%	0.93 [0.91, 0.95]
Shalom 2016	0.762	0.0019	5.6%	0.76 [0.76, 0.77]
Umezawa 2013	0.967	0.006	5.6%	0.97 [0.96, 0.98]
Van den Reek 2014b	0.85	0.0054	5.6%	0.85 [0.84, 0.86]
Vilarrasa 2016	0.772	0.003	5.6%	0.77 [0.77, 0.78]
Warren 2015	0.89	0.0007	5.6%	0.89 [0.89, 0.89]
Zweegers 2016	0.84	0.0036	5.6%	0.84 [0.83, 0.85]
**Subtotal (95% CI)**			**100.0%**	**0.82 [0.79, 0.86]**
Heterogeneity: $Tau^2 = 0.01; Chi^2 = 77443.46, df = 17 (P < 0.00001); I^2 = 100\%$				
Test for overall effect: Z = 46.06 (P < 0.00001)				
**1.5.2 Year 2**				
Arnold 2016	0.83	0.0064	6.0%	0.83 [0.82, 0.84]
Davila-Seijo 2016	0.635	0.001	6.0%	0.64 [0.63, 0.64]
García-Martínez 2015	0.71	0.013	5.9%	0.71 [0.68, 0.74]
Gniadecki 2015	0.712	0.0012	6.0%	0.71 [0.71, 0.71]
Jacobi 2016	0.546	0.0453	4.5%	0.55 [0.46, 0.63]
Marinas 2016	0.856	0.002	6.0%	0.86 [0.85, 0.86]
Menter 2016	0.815	0.0003	6.0%	0.81 [0.81, 0.82]
Menting 2014	0.627	0.022	5.6%	0.63 [0.58, 0.67]
Mercadal 2016	0.81	0.0096	5.9%	0.81 [0.79, 0.83]
Pogácsás 2017	0.724	0.0009	6.0%	0.72 [0.72, 0.73]
Richter 2017	0.375	0.0101	5.9%	0.38 [0.36, 0.39]
Ross 2016	0.932	0.0101	5.9%	0.93 [0.91, 0.95]
Shalom 2016	0.667	0.0021	6.0%	0.67 [0.66, 0.67]
Ventayol 2014	0.81	0.0096	5.9%	0.81 [0.79, 0.83]
Vilarrasa 2016	0.685	0.0033	6.0%	0.69 [0.68, 0.69]
Warren 2015	0.82	0.0009	6.0%	0.82 [0.82, 0.82]
Zweegers 2016	0.736	0.0043	6.0%	0.74 [0.73, 0.74]
**Subtotal (95% CI)**			**100.0%**	**0.73 [0.69, 0.76]**
Heterogeneity: $Tau^2 = 0.01; Chi^2 = 49428.30, df = 16 (P < 0.00001); I^2 = 100\%$				
Test for overall effect: Z = 37.49 (P < 0.00001)				
**1.5.3 Year 3**				
Arnold 2016	0.83	0.0064	6.3%	0.83 [0.82, 0.84]
Davila-Seijo 2016	0.5	0.001	6.3%	0.50 [0.50, 0.50]
García-Martínez 2015	0.69	0.0132	6.2%	0.69 [0.66, 0.72]
Gniadecki 2015	0.692	0.0012	6.3%	0.69 [0.69, 0.69]
Jacobi 2016	0.76	0.0388	5.6%	0.76 [0.68, 0.84]
Marinas 2016	0.8	0.0023	6.3%	0.80 [0.80, 0.80]
Menter 2016	0.77	0.0003	6.3%	0.77 [0.77, 0.77]
Menting 2014	0.544	0.0226	6.1%	0.54 [0.50, 0.59]
Mitratza 2017	0.478	0.02	6.1%	0.48 [0.44, 0.52]
Pogácsás 2017	0.658	0.001	6.3%	0.66 [0.66, 0.66]
Richter 2017	0.146	0.0074	6.3%	0.15 [0.13, 0.16]
Shalom 2016	0.619	0.0022	6.3%	0.62 [0.61, 0.62]
Suarez-Perez 2015	0.84	0.0083	6.3%	0.84 [0.82, 0.86]
Vilarrasa 2016	0.618	0.0035	6.3%	0.62 [0.61, 0.62]
Warren 2015	0.75	0.001	6.3%	0.75 [0.75, 0.75]
Zweegers 2016	0.712	0.0044	6.3%	0.71 [0.70, 0.72]
**Subtotal (95% CI)**			**100.0%**	**0.65 [0.60, 0.70]**
Heterogeneity: $Tau^2 = 0.01; Chi^2 = 87897.86, df = 15 (P < 0.00001); I^2 = 100\%$				
Test for overall effect: Z = 23.32 (P < 0.00001)				
**1.5.4 Year 4**				
Davila-Seijo 2016	0.423	0.001	14.3%	0.42 [0.42, 0.42]
Gniadecki 2015	0.672	0.0012	14.3%	0.67 [0.67, 0.67]
Marinas 2016	0.755	0.0024	14.3%	0.76 [0.75, 0.76]
Menter 2016	0.757	0.0003	14.3%	0.76 [0.76, 0.76]
Richter 2017	0.042	0.0042	14.3%	0.04 [0.03, 0.05]
Vilarrasa 2016	0.578	0.0035	14.3%	0.58 [0.57, 0.58]
Zweegers 2016	0.712	0.0044	14.3%	0.71 [0.70, 0.72]
**Subtotal (95% CI)**			**100.0%**	**0.56 [0.42, 0.70]**
Heterogeneity: $Tau^2 = 0.04; Chi^2 = 132840.63, df = 6 (P < 0.00001); I^2 = 100\%$				
Test for overall effect: Z = 7.83 (P < 0.00001)				

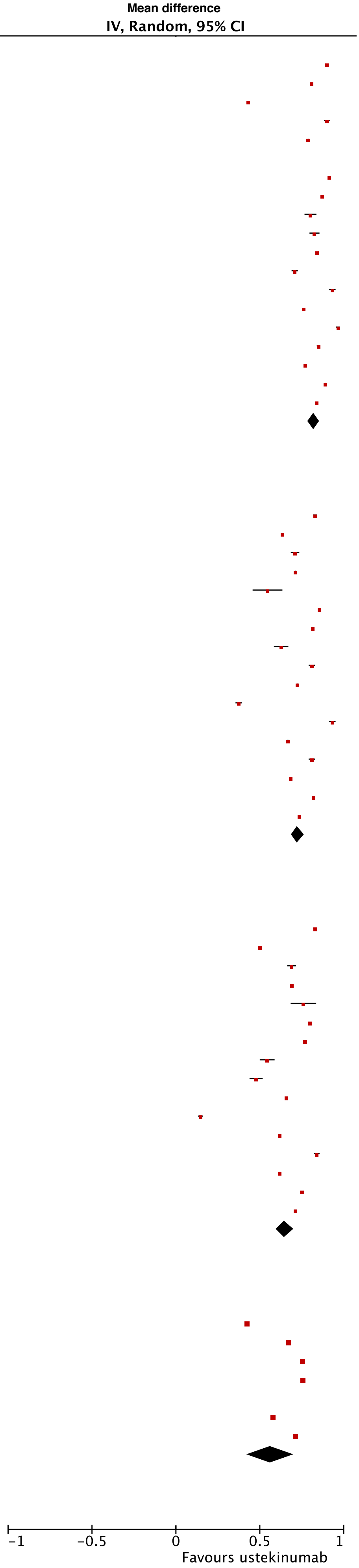

Test for subgroup differences: $Chi^2 = 37.57, df = 3 (P < 0.00001), I^2 = 92.0\%$

Supplementary Figure S2(a): Forest plots of drug survival of biologics for treating psoriasis in biologic-naïve subjects with etanercept.

Study or Subgroup	Risk Difference	SE	Weight	Mean difference IV, Random, 95% CI
**2.1.1 Year 1**				
Bonafeda 2013	0.485	0.0003	16.7%	0.48 [0.48, 0.49]
Brunasso 2012	0.62	0.0143	16.6%	0.62 [0.59, 0.65]
Menter 2016	0.677	0.0016	16.7%	0.68 [0.67, 0.68]
Menting 2014	0.85	0.003	16.7%	0.85 [0.84, 0.86]
Mitratza 2017	0.64	0.0083	16.7%	0.64 [0.62, 0.66]
Van den Reek 2014a	0.718	0.003	16.7%	0.72 [0.71, 0.72]
**Subtotal (95% CI)**			**100.0%**	**0.67 [0.53, 0.80]**

Heterogeneity: $Tau^2 = 0.03$; $Chi^2 = 33898.29$, df = 5 ($P < 0.00001$); $I^2 = 100\%$

Test for overall effect: Z = 9.75 ($P < 0.00001$)

Study or Subgroup	Risk Difference	SE	Weight	Mean difference IV, Random, 95% CI
**2.1.2 Year 2**				
Brunasso 2012	0.62	0.0143	19.8%	0.62 [0.59, 0.65]
Menter 2016	0.581	0.0017	20.1%	0.58 [0.58, 0.58]
Menting 2014	0.796	0.0034	20.1%	0.80 [0.79, 0.80]
Van den Reek 2014a	0.577	0.0033	20.1%	0.58 [0.57, 0.58]
Ventayol 2014	0.62	0.0143	19.8%	0.62 [0.59, 0.65]
**Subtotal (95% CI)**			**100.0%**	**0.64 [0.54, 0.74]**

Heterogeneity: $Tau^2 = 0.01$; $Chi^2 = 3366.46$, df = 4 ($P < 0.00001$); $I^2 = 100\%$

Test for overall effect: Z = 12.99 ($P < 0.00001$)

Study or Subgroup	Risk Difference	SE	Weight	Mean difference IV, Random, 95% CI
**2.1.3 Year 3**				
Brunasso 2012	0.62	0.0143	19.9%	0.62 [0.59, 0.65]
Menter 2016	0.532	0.0017	20.1%	0.53 [0.53, 0.54]
Menting 2014	0.77	0.0036	20.0%	0.77 [0.76, 0.78]
Mitratza 2017	0.26	0.0076	20.0%	0.26 [0.25, 0.27]
Van den Reek 2014a	0.468	0.0034	20.0%	0.47 [0.46, 0.47]
**Subtotal (95% CI)**			**100.0%**	**0.53 [0.40, 0.66]**

Heterogeneity: $Tau^2 = 0.02$; $Chi^2 = 5906.35$, df = 4 ($P < 0.00001$); $I^2 = 100\%$

Test for overall effect: Z = 8.16 ($P < 0.00001$)

Study or Subgroup	Risk Difference	SE	Weight	Mean difference IV, Random, 95% CI
**2.1.4 Year 4**				
Brunasso 2012	0.62	0.0106	24.9%	0.62 [0.60, 0.64]
Menter 2016	0.484	0.0017	25.1%	0.48 [0.48, 0.49]
Menting 2014	0.64	0.0041	25.0%	0.64 [0.63, 0.65]
Van den Reek 2014a	0.346	0.0032	25.0%	0.35 [0.34, 0.35]
**Subtotal (95% CI)**			**100.0%**	**0.52 [0.41, 0.64]**

Heterogeneity: $Tau^2 = 0.01$; $Chi^2 = 3454.68$, df = 3 ($P < 0.00001$); $I^2 = 100\%$

Test for overall effect: Z = 8.92 ($P < 0.00001$)

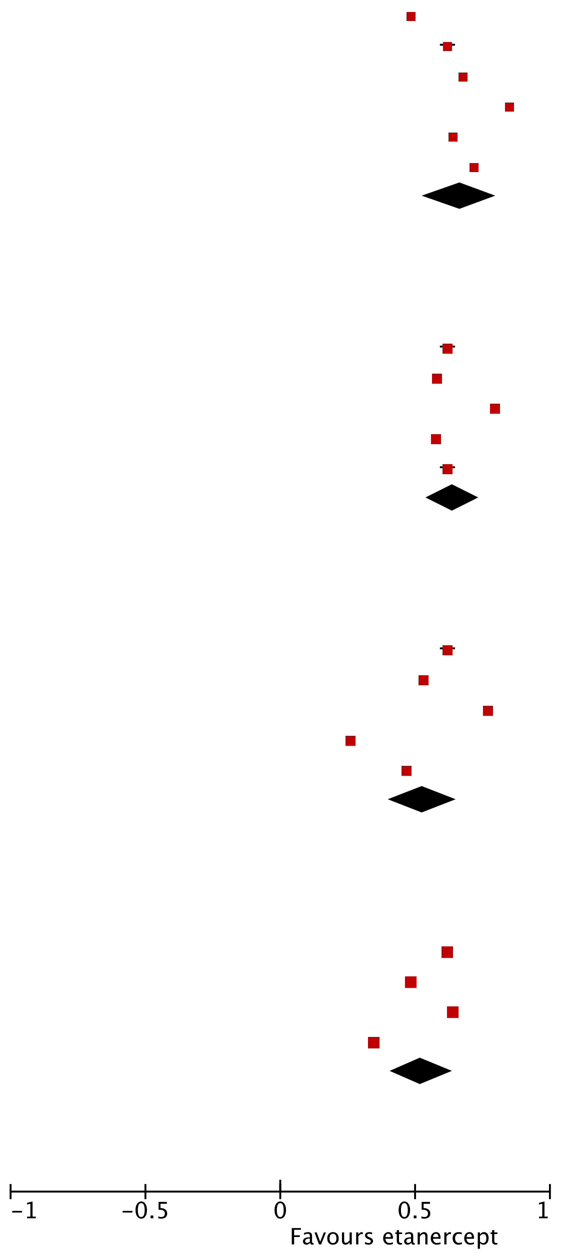

Test for subgroup differences: $Chi^2 = 4.39$, df = 3 ($P = 0.22$), $I^2 = 31.6\%$

Supplementary Figure S2(b): Forest plots of drug survival of biologics for treating psoriasis in biologic-naïve subjects with adalimumab.

Study or Subgroup	Risk Difference	SE	Weight	Mean difference IV, Random, 95% CI
**2.2.1 Year 1**				
Bonafeda 2013	0.492	0.0003	20.1%	0.49 [0.49, 0.49]
Brunasso 2012	1	0		Not estimable
Lopez–Ferrer 2013	0.785	0.0084	20.0%	0.79 [0.77, 0.80]
Menter 2016	0.792	0.001	20.0%	0.79 [0.79, 0.79]
Menting 2014	0.77	0.0145	20.0%	0.77 [0.74, 0.80]
Mitratza 2017	0.519	0.0167	19.9%	0.52 [0.49, 0.55]
**Subtotal (95% CI)**			**100.0%**	**0.67 [0.49, 0.86]**
Heterogeneity: $Tau^2 = 0.04$; $Chi^2 = 83891.40$, df = 4 ($P < 0.00001$); $I^2 = 100\%$				
Test for overall effect: Z = 7.09 ($P < 0.00001$)				
**2.2.2 Year 2**				
Brunasso 2012	1	0		Not estimable
Lopez–Ferrer 2013	0.785	0.0084	25.1%	0.79 [0.77, 0.80]
Menter 2016	0.655	0.0012	25.3%	0.66 [0.65, 0.66]
Menting 2014	0.77	0.0145	24.8%	0.77 [0.74, 0.80]
Ventayol 2014	0.48	0.0135	24.8%	0.48 [0.45, 0.51]
**Subtotal (95% CI)**			**100.0%**	**0.67 [0.58, 0.77]**
Heterogeneity: $Tau^2 = 0.01$; $Chi^2 = 467.61$, df = 3 ($P < 0.00001$); $I^2 = 99\%$				
Test for overall effect: Z = 13.54 ($P < 0.00001$)				
**2.2.3 Year 3**				
Brunasso 2012	1	0		Not estimable
Lopez–Ferrer 2013	0.785	0.0084	25.0%	0.79 [0.77, 0.80]
Menter 2016	0.655	0.0012	25.1%	0.66 [0.65, 0.66]
Menting 2014	0.77	0.0145	24.9%	0.77 [0.74, 0.80]
Mitratza 2017	0.222	0.0139	24.9%	0.22 [0.19, 0.25]
**Subtotal (95% CI)**			**100.0%**	**0.61 [0.45, 0.77]**
Heterogeneity: $Tau^2 = 0.03$; $Chi^2 = 1272.77$, df = 3 ($P < 0.00001$); $I^2 = 100\%$				
Test for overall effect: Z = 7.39 ($P < 0.00001$)				
**2.2.4 Year 4**				
Brunasso 2012	1	0		Not estimable
Lopez–Ferrer 2013	0.659	0.0097	33.3%	0.66 [0.64, 0.68]
Menter 2016	0.468	0.0012	33.5%	0.47 [0.47, 0.47]
Menting 2014	0.77	0.0145	33.2%	0.77 [0.74, 0.80]
**Subtotal (95% CI)**			**100.0%**	**0.63 [0.45, 0.82]**
Heterogeneity: $Tau^2 = 0.03$; $Chi^2 = 804.59$, df = 2 ($P < 0.00001$); $I^2 = 100\%$				
Test for overall effect: Z = 6.71 ($P < 0.00001$)				

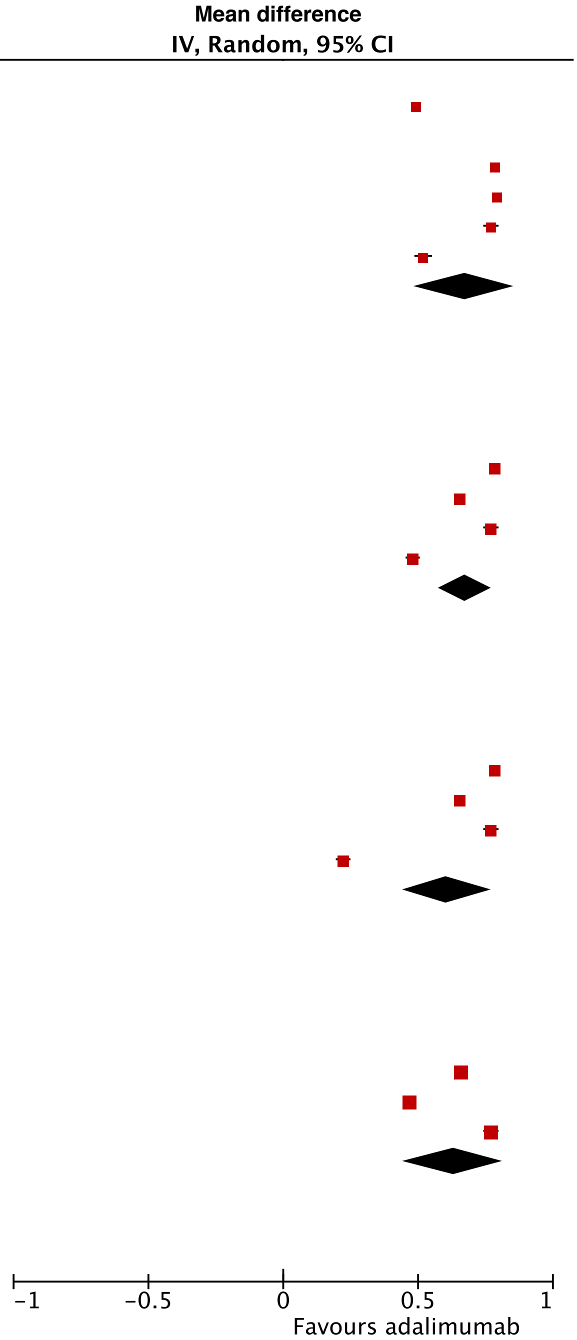

Test for subgroup differences: $Chi^2 = 0.54$, df = 3 ($P = 0.91$), $I^2 = 0\%$

Supplementary Figure S2(c): Forest plots of drug survival of biologics for treating psoriasis in biologic-naïve subjects with infliximab.

Study or Subgroup	Risk Difference	SE	Weight	Mean difference IV, Random, 95% CI
**2.3.1 Year 1**				
Brunasso 2012	0.18	0.0116	25.0%	0.18 [0.16, 0.20]
Menter 2016	0.839	0.0058	25.0%	0.84 [0.83, 0.85]
Menting 2014	0.75	0.0228	25.0%	0.75 [0.71, 0.79]
Mitratza 2017	0.524	0.0238	25.0%	0.52 [0.48, 0.57]
**Subtotal (95% CI)**			**100.0%**	**0.57 [0.19, 0.95]**
Heterogeneity: $Tau^2 = 0.15$; $Chi^2 = 2643.28$, df = 3 ($P < 0.00001$); $I^2 = 100\%$				
Test for overall effect: Z = 2.96 (P = 0.003)				
**2.3.2 Year 2**				
Brunasso 2012	0.14	0.0105	33.4%	0.14 [0.12, 0.16]
Menter 2016	0.774	0.0066	33.4%	0.77 [0.76, 0.79]
Menting 2014	0.75	0.0228	33.3%	0.75 [0.71, 0.79]
**Subtotal (95% CI)**			**100.0%**	**0.55 [0.09, 1.02]**
Heterogeneity: $Tau^2 = 0.17$; $Chi^2 = 2657.24$, df = 2 ($P < 0.00001$); $I^2 = 100\%$				
Test for overall effect: Z = 2.35 (P = 0.02)				
**2.3.3 Year 3**				
Brunasso 2012	0.14	0.0105	25.0%	0.14 [0.12, 0.16]
Menter 2016	0.677	0.0074	25.1%	0.68 [0.66, 0.69]
Menting 2014	0.75	0.0228	25.0%	0.75 [0.71, 0.79]
Mitratza 2017	0.429	0.0236	24.9%	0.43 [0.38, 0.48]
**Subtotal (95% CI)**			**100.0%**	**0.50 [0.18, 0.82]**
Heterogeneity: $Tau^2 = 0.11$; $Chi^2 = 1873.60$, df = 3 ($P < 0.00001$); $I^2 = 100\%$				
Test for overall effect: Z = 3.04 (P = 0.002)				
**2.3.4 Year 4**				
Brunasso 2012	0.14	0.0105	33.4%	0.14 [0.12, 0.16]
Menter 2016	0.532	0.0079	33.4%	0.53 [0.52, 0.55]
Menting 2014	0.75	0.0228	33.2%	0.75 [0.71, 0.79]
**Subtotal (95% CI)**			**100.0%**	**0.47 [0.16, 0.79]**
Heterogeneity: $Tau^2 = 0.08$; $Chi^2 = 1121.14$, df = 2 ($P < 0.00001$); $I^2 = 100\%$				
Test for overall effect: Z = 2.93 (P = 0.003)				

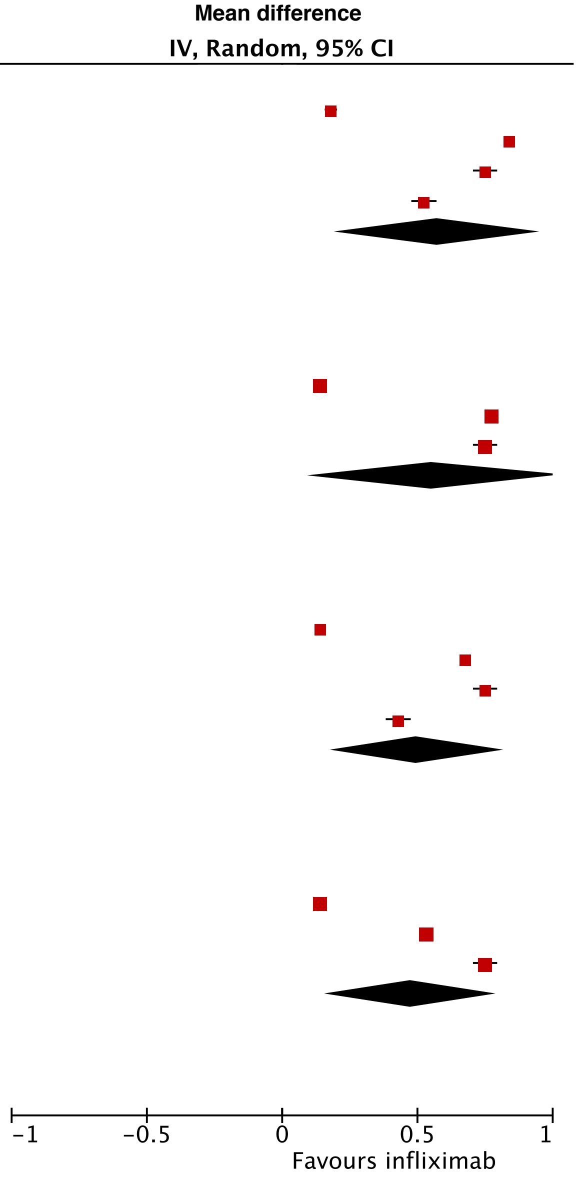

Test for subgroup differences: $Chi^2 = 0.19$, df = 3 (P = 0.98), $I^2 = 0\%$

Supplemetnary Figure S2(d): Forest plots of drug survival of biologics for treating psoriasis in biologic-naïve subjects with ustekinumab.

Study or Subgroup	Risk Difference	SE	Weight	Mean difference IV, Random, 95% CI
**2.4.1 Year 1**				
Menter 2016	0.936	0.0008	51.0%	0.94 [0.93, 0.94]
Mitratza 2017	0.826	0.0152	49.0%	0.83 [0.80, 0.86]
**Subtotal (95% CI)**			**100.0%**	**0.88 [0.77, 0.99]**
Heterogeneity: $Tau^2 = 0.01$; $Chi^2 = 52.23$, df = 1 (P < 0.00001); $I^2 = 98\%$				
Test for overall effect: Z = 16.04 (P < 0.00001)				
**2.4.2 Year 2**				
Menter 2016	0.903	0.001	50.5%	0.90 [0.90, 0.90]
Ventayol 2014	0.81	0.0096	49.5%	0.81 [0.79, 0.83]
**Subtotal (95% CI)**			**100.0%**	**0.86 [0.77, 0.95]**
Heterogeneity: $Tau^2 = 0.00$; $Chi^2 = 92.84$, df = 1 (P < 0.00001); $I^2 = 99\%$				
Test for overall effect: Z = 18.43 (P < 0.00001)				
**2.4.3 Year 3**				
Menter 2016	0.871	0.0011	50.1%	0.87 [0.87, 0.87]
Mitratza 2017	0.478	0.02	49.9%	0.48 [0.44, 0.52]
**Subtotal (95% CI)**			**100.0%**	**0.68 [0.29, 1.06]**
Heterogeneity: $Tau^2 = 0.08$; $Chi^2 = 384.96$, df = 1 (P < 0.00001); $I^2 = 100\%$				
Test for overall effect: Z = 3.44 (P = 0.0006)				
**2.4.4 Year 4**				
Menter 2016	0.871	0.0011	100.0%	0.87 [0.87, 0.87]
**Subtotal (95% CI)**			**100.0%**	**0.87 [0.87, 0.87]**
Heterogeneity: Not applicable				
Test for overall effect: Z = 791.82 (P < 0.00001)				

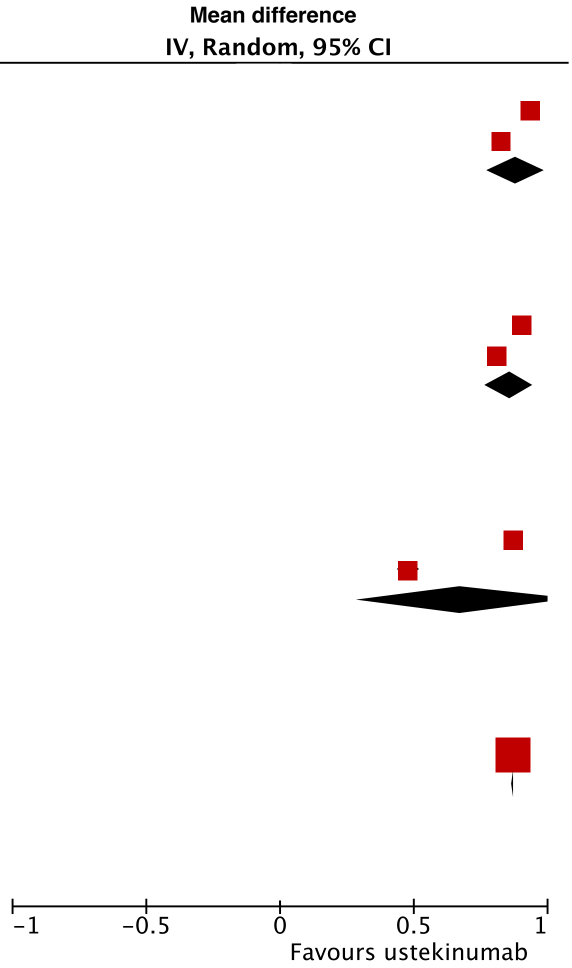

Test for subgroup differences: $Chi^2 = 1.13$, df = 3 (P = 0.77), $I^2 = 0\%$

Supplemetnary Figure S3(a): Forest plots of drug survival for discontinue due to loss of efficacy with etanercept.

Study or Subgroup	Risk Difference	SE	Weight	Mean difference IV, Random, 95% CI
**3.1.1 Year 1**				
Davila-Seijo 2016	0.808	0.0006	21.0%	0.81 [0.81, 0.81]
Van den Reek 2014b	0.83	0.0046	16.9%	0.83 [0.82, 0.84]
Vilarrasa 2016	0.776	0.0017	20.4%	0.78 [0.77, 0.78]
Warren 2015	0.8	0.0004	21.1%	0.80 [0.80, 0.80]
Zweegers 2016	0.811	0.0016	20.5%	0.81 [0.81, 0.81]
**Subtotal (95% CI)**			**100.0%**	**0.80 [0.80, 0.81]**
Heterogeneity: $Tau^2 = 0.00$; $Chi^2 = 427.60$, df = 4 ($P < 0.00001$); $I^2 = 99\%$				
Test for overall effect: Z = 190.32 ($P < 0.00001$)				
**3.1.2 Year 2**				
Davila-Seijo 2016	0.654	0.0007	25.1%	0.65 [0.65, 0.66]
Vilarrasa 2016	0.571	0.002	24.9%	0.57 [0.57, 0.57]
Warren 2015	0.65	0.0004	25.1%	0.65 [0.65, 0.65]
Zweegers 2016	0.676	0.002	24.9%	0.68 [0.67, 0.68]
**Subtotal (95% CI)**			**100.0%**	**0.64 [0.62, 0.66]**
Heterogeneity: $Tau^2 = 0.00$; $Chi^2 = 1758.96$, df = 3 ($P < 0.00001$); $I^2 = 100\%$				
Test for overall effect: Z = 59.66 ($P < 0.00001$)				
**3.1.3 Year 3**				
Davila-Seijo 2016	0.539	0.0007	25.0%	0.54 [0.54, 0.54]
Vilarrasa 2016	0.469	0.002	24.9%	0.47 [0.47, 0.47]
Warren 2015	0.55	0.0005	25.1%	0.55 [0.55, 0.55]
Zweegers 2016	0.622	0.002	24.9%	0.62 [0.62, 0.63]
**Subtotal (95% CI)**			**100.0%**	**0.54 [0.52, 0.57]**
Heterogeneity: $Tau^2 = 0.00$; $Chi^2 = 3089.92$, df = 3 ($P < 0.00001$); $I^2 = 100\%$				
Test for overall effect: Z = 36.34 ($P < 0.00001$)				
**3.1.4 Year 4**				
Davila-Seijo 2016	0.5	0.0007	33.4%	0.50 [0.50, 0.50]
Vilarrasa 2016	0.408	0.002	33.3%	0.41 [0.40, 0.41]
Zweegers 2016	0.514	0.0021	33.3%	0.51 [0.51, 0.52]
**Subtotal (95% CI)**			**100.0%**	**0.47 [0.42, 0.53]**
Heterogeneity: $Tau^2 = 0.00$; $Chi^2 = 2004.32$, df = 2 ($P < 0.00001$); $I^2 = 100\%$				
Test for overall effect: Z = 16.58 ($P < 0.00001$)				

Mean difference
IV, Random, 95% CI

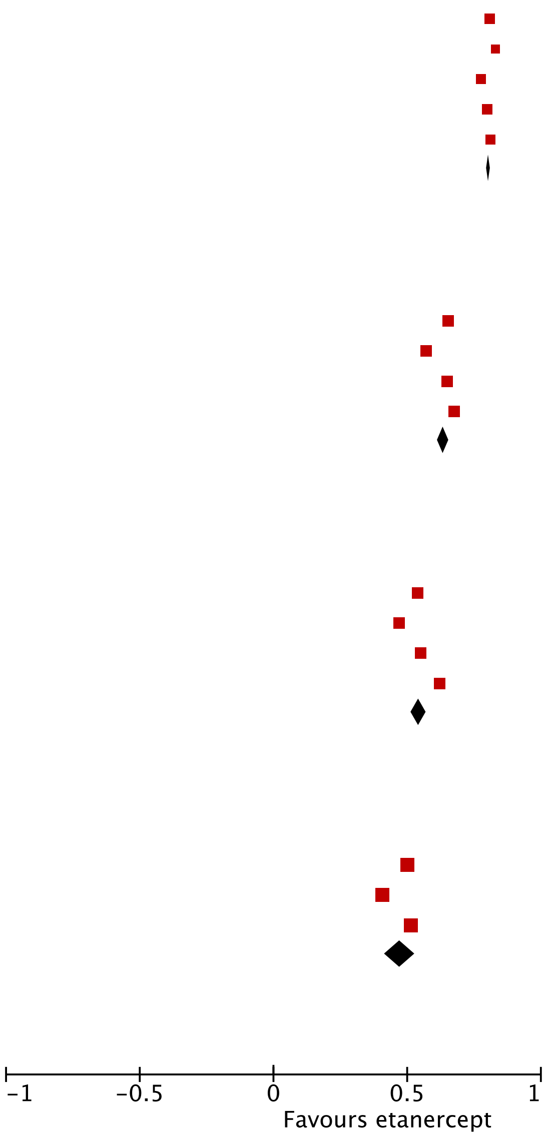

Test for subgroup differences: $Chi^2 = 545.09$, df = 3 ($P < 0.00001$), $I^2 = 99.4\%$

Supplemetnary Figure S3(b): Forest plots of drug survival for discontinue due to loss of efficacy with adalimumab.

Study or Subgroup	Risk Difference	SE	Weight	Mean difference IV, Random, 95% CI
**3.2.1 Year 1**				
Davila-Seijo 2016	0.962	0.0003	20.0%	0.96 [0.96, 0.96]
Van den Reek 2014b	0.85	0.0035	19.9%	0.85 [0.84, 0.86]
Vilarrasa 2016	0.816	0.0017	20.0%	0.82 [0.81, 0.82]
Warren 2015	0.9	0.0002	20.0%	0.90 [0.90, 0.90]
Zweegers 2016	0.865	0.0018	20.0%	0.86 [0.86, 0.87]
**Subtotal (95% CI)**			**100.0%**	**0.88 [0.84, 0.92]**
Heterogeneity: $Tau^2 = 0.00; Chi^2 = 34446.45, df = 4 (P < 0.00001); I^2 = 100\%$				
Test for overall effect: Z = 43.00 (P < 0.00001)				
**3.2.2 Year 2**				
Davila-Seijo 2016	0.673	0.0006	25.0%	0.67 [0.67, 0.67]
Vilarrasa 2016	0.673	0.002	25.0%	0.67 [0.67, 0.68]
Warren 2015	0.83	0.0002	25.0%	0.83 [0.83, 0.83]
Zweegers 2016	0.73	0.0024	25.0%	0.73 [0.73, 0.73]
**Subtotal (95% CI)**			**100.0%**	**0.73 [0.62, 0.83]**
Heterogeneity: $Tau^2 = 0.01; Chi^2 = 67759.18, df = 3 (P < 0.00001); I^2 = 100\%$				
Test for overall effect: Z = 13.40 (P < 0.00001)				
**3.2.3 Year 3**				
Davila-Seijo 2016	0.615	0.0007	25.0%	0.61 [0.61, 0.62]
Vilarrasa 2016	0.612	0.0021	25.0%	0.61 [0.61, 0.62]
Warren 2015	0.79	0.0002	25.0%	0.79 [0.79, 0.79]
Zweegers 2016	0.649	0.0026	25.0%	0.65 [0.64, 0.65]
**Subtotal (95% CI)**			**100.0%**	**0.67 [0.55, 0.79]**
Heterogeneity: $Tau^2 = 0.01; Chi^2 = 66241.28, df = 3 (P < 0.00001); I^2 = 100\%$				
Test for overall effect: Z = 10.94 (P < 0.00001)				
**3.2.4 Year 4**				
Davila-Seijo 2016	0.539	0.0007	25.0%	0.54 [0.54, 0.54]
Van den Reek 2016	0.62	0.0011	25.0%	0.62 [0.62, 0.62]
Vilarrasa 2016	0.51	0.0022	25.0%	0.51 [0.51, 0.51]
Zweegers 2016	0.622	0.0026	25.0%	0.62 [0.62, 0.63]
**Subtotal (95% CI)**			**100.0%**	**0.57 [0.52, 0.63]**
Heterogeneity: $Tau^2 = 0.00; Chi^2 = 4950.74, df = 3 (P < 0.00001); I^2 = 100\%$				
Test for overall effect: Z = 21.20 (P < 0.00001)				

Mean difference IV, Random, 95% CI

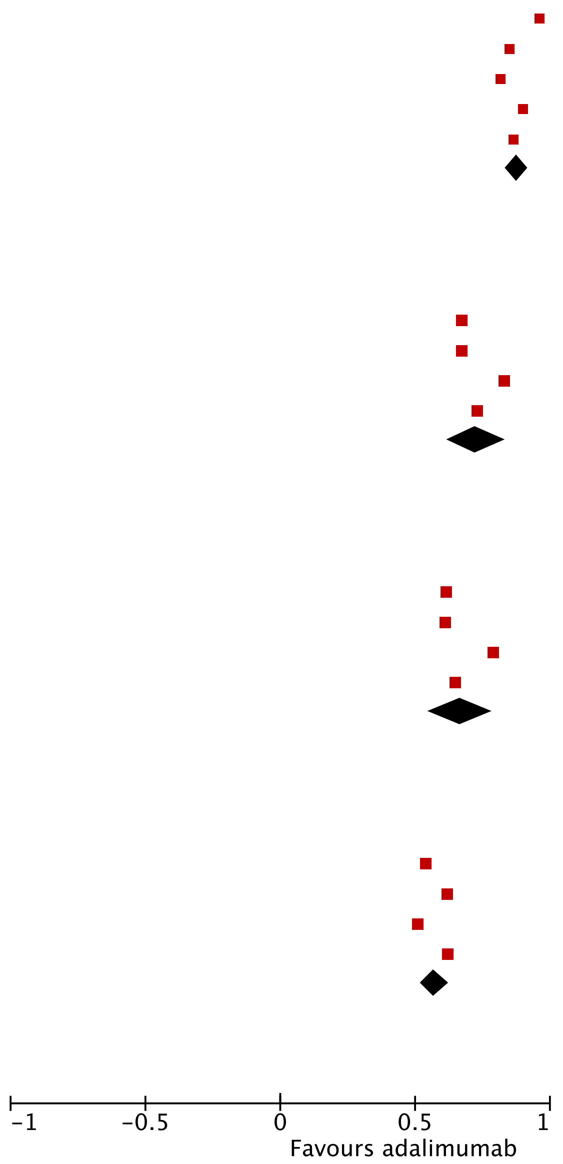

Test for subgroup differences: $Chi^2 = 84.49, df = 3 (P < 0.00001), I^2 = 96.4\%$

Supplemetnary Figure S3(c): Forest plots of drug survival for discontinue due to loss of efficacy with infliximab.

Study or Subgroup	Risk Difference	SE	Weight	Mean difference IV, Random, 95% CI
**3.3.1 Year 1**				
Davila-Seijo 2016	0.962	0.001	33.4%	0.96 [0.96, 0.96]
Vilarrasa 2016	0.796	0.0048	33.3%	0.80 [0.79, 0.81]
Warren 2015	0.86	0.0036	33.3%	0.86 [0.85, 0.87]
**Subtotal (95% CI)**			**100.0%**	**0.87 [0.77, 0.97]**
Heterogeneity: Tau^2 = 0.01; Chi^2 = 1795.97, df = 2 (P < 0.00001); I^2 = 100%				
Test for overall effect: Z = 16.77 (P < 0.00001)				
**3.3.2 Year 2**				
Davila-Seijo 2016	0.692	0.0025	33.4%	0.69 [0.69, 0.70]
Vilarrasa 2016	0.653	0.0057	33.2%	0.65 [0.64, 0.66]
Warren 2015	0.79	0.0042	33.3%	0.79 [0.78, 0.80]
**Subtotal (95% CI)**			**100.0%**	**0.71 [0.64, 0.78]**
Heterogeneity: Tau^2 = 0.00; Chi^2 = 514.61, df = 2 (P < 0.00001); I^2 = 100%				
Test for overall effect: Z = 19.37 (P < 0.00001)				
**3.3.3 Year 3**				
Davila-Seijo 2016	0.577	0.0026	33.4%	0.58 [0.57, 0.58]
Vilarrasa 2016	0.571	0.0059	33.3%	0.57 [0.56, 0.58]
Warren 2015	0.76	0.0044	33.3%	0.76 [0.75, 0.77]
**Subtotal (95% CI)**			**100.0%**	**0.64 [0.51, 0.76]**
Heterogeneity: Tau^2 = 0.01; Chi^2 = 1353.63, df = 2 (P < 0.00001); I^2 = 100%				
Test for overall effect: Z = 10.25 (P < 0.00001)				
**3.3.4 Year 4**				
Davila-Seijo 2016	0.539	0.0027	50.6%	0.54 [0.53, 0.54]
Vilarrasa 2016	0.49	0.006	49.4%	0.49 [0.48, 0.50]
**Subtotal (95% CI)**			**100.0%**	**0.51 [0.47, 0.56]**
Heterogeneity: Tau^2 = 0.00; Chi^2 = 55.46, df = 1 (P < 0.00001); I^2 = 98%				
Test for overall effect: Z = 21.01 (P < 0.00001)				

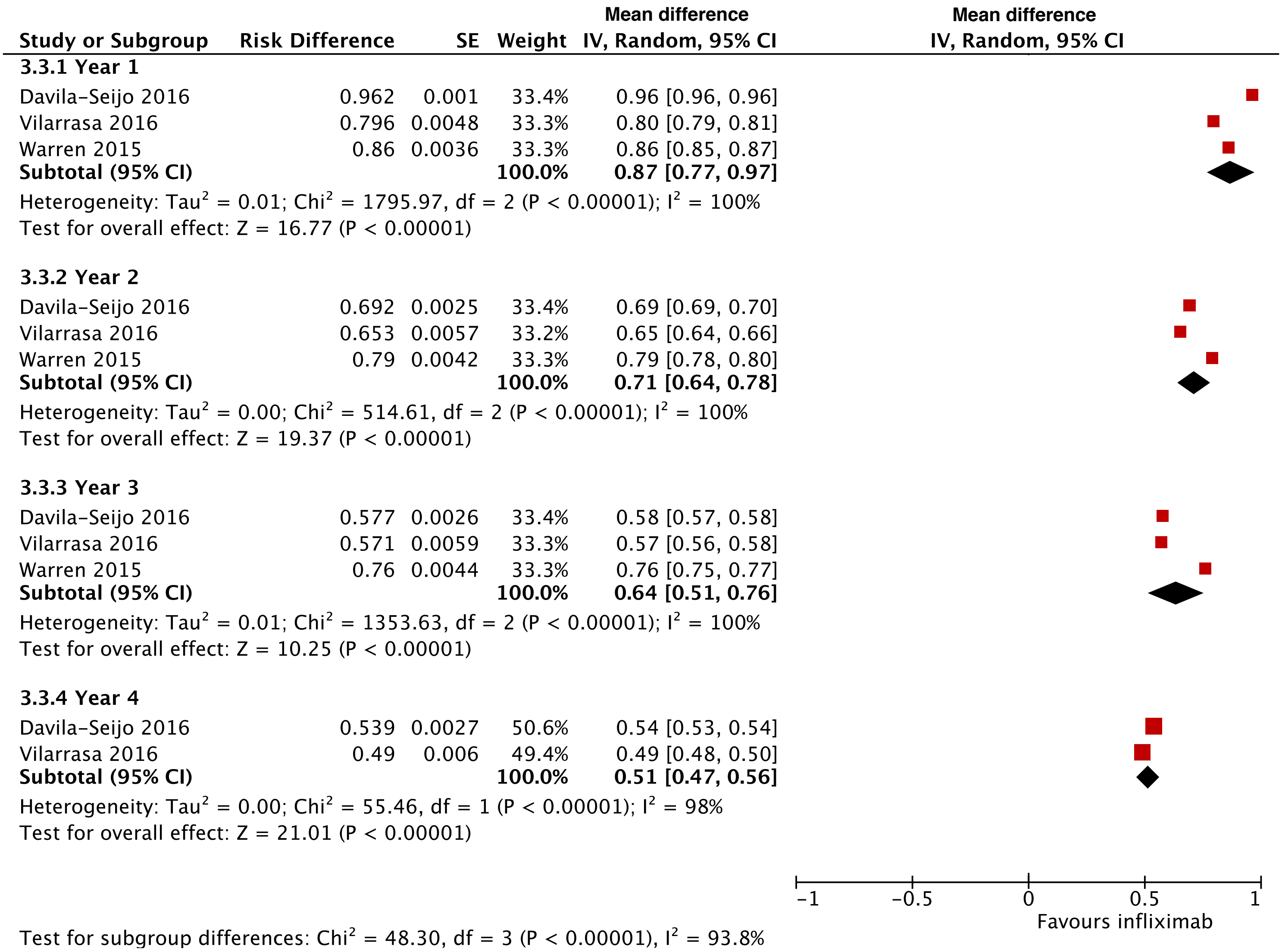

Test for subgroup differences: Chi^2 = 48.30, df = 3 (P < 0.00001), I^2 = 93.8%

Supplemetnary Figure S3(d): Forest plots of drug survival for discontinue due to loss of efficacy with ustekinumab.

Study or Subgroup	Risk Difference	SE	Weight	Mean difference IV, Random, 95% CI
**3.4.1 Year 1**				
Davila–Seijo 2016	0.904	0.0006	20.1%	0.90 [0.90, 0.91]
Van den Reek 2014b	0.94	0.0036	19.9%	0.94 [0.93, 0.95]
Vilarrasa 2016	0.938	0.0017	20.0%	0.94 [0.93, 0.94]
Warren 2015	0.96	0.0004	20.1%	0.96 [0.96, 0.96]
Zweegers 2016	0.919	0.0027	20.0%	0.92 [0.91, 0.92]
**Subtotal (95% CI)**			**100.0%**	**0.93 [0.90, 0.96]**
Heterogeneity: $Tau^2 = 0.00$; $Chi^2 = 6113.99$, df = 4 ($P < 0.00001$); $I^2 = 100\%$				
Test for overall effect: $Z = 57.67$ ($P < 0.00001$)				
**3.4.2 Year 2**				
Davila–Seijo 2016	0.846	0.0007	25.0%	0.85 [0.84, 0.85]
Vilarrasa 2016	0.938	0.0017	25.0%	0.94 [0.93, 0.94]
Warren 2015	0.93	0.0006	25.0%	0.93 [0.93, 0.93]
Zweegers 2016	0.865	0.0034	24.9%	0.86 [0.86, 0.87]
**Subtotal (95% CI)**			**100.0%**	**0.89 [0.84, 0.95]**
Heterogeneity: $Tau^2 = 0.00$; $Chi^2 = 9003.22$, df = 3 ($P < 0.00001$); $I^2 = 100\%$				
Test for overall effect: $Z = 32.46$ ($P < 0.00001$)				
**3.4.3 Year 3**				
Davila–Seijo 2016	0.769	0.0009	25.0%	0.77 [0.77, 0.77]
Vilarrasa 2016	0.857	0.0025	25.0%	0.86 [0.85, 0.86]
Warren 2015	0.89	0.0007	25.0%	0.89 [0.89, 0.89]
Zweegers 2016	0.865	0.0034	25.0%	0.86 [0.86, 0.87]
**Subtotal (95% CI)**			**100.0%**	**0.85 [0.77, 0.92]**
Heterogeneity: $Tau^2 = 0.01$; $Chi^2 = 11320.38$, df = 3 ($P < 0.00001$); $I^2 = 100\%$				
Test for overall effect: $Z = 21.88$ ($P < 0.00001$)				
**3.4.4 Year 4**				
Davila–Seijo 2016	0.731	0.0009	33.4%	0.73 [0.73, 0.73]
Vilarrasa 2016	0.816	0.0028	33.3%	0.82 [0.81, 0.82]
Zweegers 2016	0.838	0.0036	33.3%	0.84 [0.83, 0.85]
**Subtotal (95% CI)**			**100.0%**	**0.79 [0.72, 0.87]**
Heterogeneity: $Tau^2 = 0.00$; $Chi^2 = 1551.55$, df = 2 ($P < 0.00001$); $I^2 = 100\%$				
Test for overall effect: $Z = 21.11$ ($P < 0.00001$)				

Mean difference
IV, Random, 95% CI

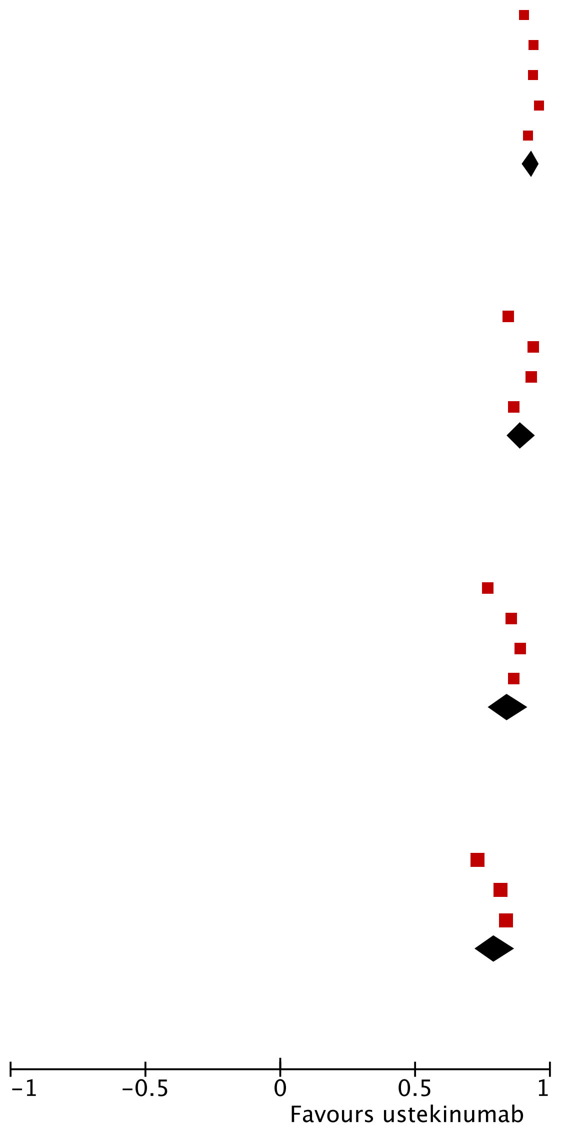

Test for subgroup differences: $Chi^2 = 13.79$, df = 3 ($P = 0.003$), $I^2 = 78.2\%$

Supplemetnary Figure S4(a): Forest plots of drug survival for discontinue due to adverse effects with etanercept.

Study or Subgroup	Risk Difference	SE	Weight	Mean difference IV, Random, 95% CI
**4.1.1 Year 1**				
Davila-Seijo 2016	0.923	0.0004	25.5%	0.92 [0.92, 0.92]
Van den Reek 2014b	0.9	0.0037	23.7%	0.90 [0.89, 0.91]
Warren 2015	0.94	0.0002	25.5%	0.94 [0.94, 0.94]
Zweegers 2016	0.959	0.0008	25.4%	0.96 [0.96, 0.96]
**Subtotal (95% CI)**			**100.0%**	**0.93 [0.92, 0.94]**
Heterogeneity: $Tau^2 = 0.00$; $Chi^2 = 2295.07$, df = 3 (P < 0.00001); $I^2 = 100\%$				
Test for overall effect: Z = 136.86 (P < 0.00001)				
**4.1.2 Year 2**				
Davila-Seijo 2016	0.885	0.0005	33.4%	0.89 [0.88, 0.89]
Warren 2015	0.91	0.0003	33.4%	0.91 [0.91, 0.91]
Zweegers 2016	0.892	0.0013	33.2%	0.89 [0.89, 0.89]
**Subtotal (95% CI)**			**100.0%**	**0.90 [0.88, 0.91]**
Heterogeneity: $Tau^2 = 0.00$; $Chi^2 = 1912.01$, df = 2 (P < 0.00001); $I^2 = 100\%$				
Test for overall effect: Z = 92.45 (P < 0.00001)				
**4.1.3 Year 3**				
Davila-Seijo 2016	0.846	0.0005	33.3%	0.85 [0.85, 0.85]
Warren 2015	0.9	0.0003	33.3%	0.90 [0.90, 0.90]
Zweegers 2016	0.892	0.0013	33.3%	0.89 [0.89, 0.89]
**Subtotal (95% CI)**			**100.0%**	**0.88 [0.84, 0.92]**
Heterogeneity: $Tau^2 = 0.00$; $Chi^2 = 8599.03$, df = 2 (P < 0.00001); $I^2 = 100\%$				
Test for overall effect: Z = 42.82 (P < 0.00001)				
**4.1.4 Year 4**				
Davila-Seijo 2016	0.888	0.0005	54.5%	0.89 [0.89, 0.89]
Zweegers 2016	0.892	0.0013	45.5%	0.89 [0.89, 0.89]
**Subtotal (95% CI)**			**100.0%**	**0.89 [0.89, 0.89]**
Heterogeneity: $Tau^2 = 0.00$; $Chi^2 = 8.25$, df = 1 (P = 0.004); $I^2 = 88\%$				
Test for overall effect: Z = 446.72 (P < 0.00001)				

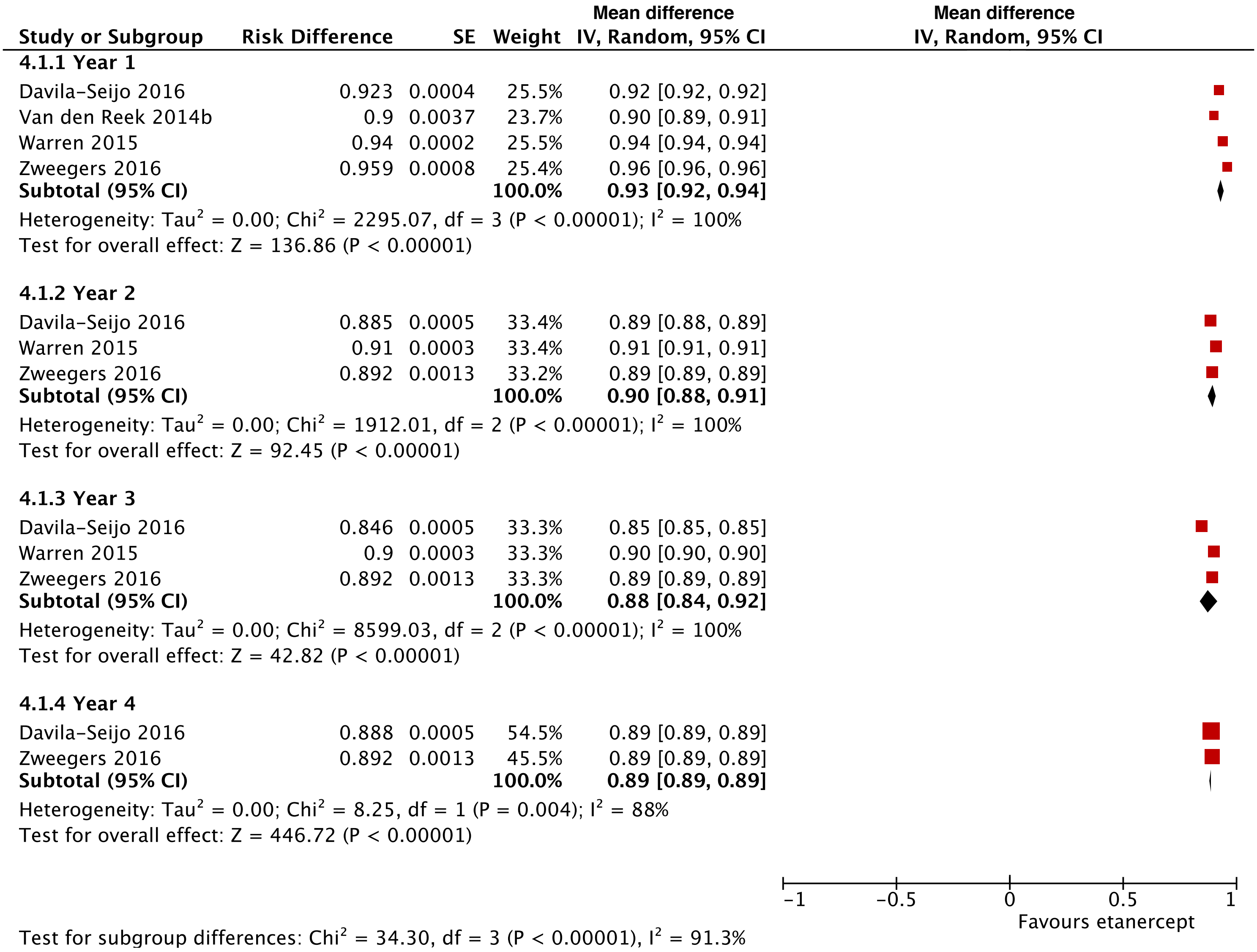

Test for subgroup differences: $Chi^2 = 34.30$, df = 3 (P < 0.00001), $I^2 = 91.3\%$

Supplemetnary Figure S4(b): Forest plots of drug survival for discontinue due to adverse effects with adalimumab.

Study or Subgroup	Risk Difference	SE	Weight	Mean difference IV, Random, 95% CI
**4.2.1 Year 1**				
Davila-Seijo 2016	0.923	0.0004	25.7%	0.92 [0.92, 0.92]
Van den Reek 2014b	0.95	0.0022	24.0%	0.95 [0.95, 0.95]
Warren 2015	0.93	0.0001	25.7%	0.93 [0.93, 0.93]
Zweegers 2016	0.892	0.0017	24.7%	0.89 [0.89, 0.90]
**Subtotal (95% CI)**			**100.0%**	**0.92 [0.92, 0.93]**
Heterogeneity: Tau^2 = 0.00; Chi^2 = 862.48, df = 3 (P < 0.00001); I^2 = 100%				
Test for overall effect: Z = 222.32 (P < 0.00001)				
**4.2.2 Year 2**				
Davila-Seijo 2016	0.885	0.0004	33.5%	0.89 [0.88, 0.89]
Warren 2015	0.9	0.0002	33.6%	0.90 [0.90, 0.90]
Zweegers 2016	0.838	0.002	32.9%	0.84 [0.83, 0.84]
**Subtotal (95% CI)**			**100.0%**	**0.87 [0.86, 0.89]**
Heterogeneity: Tau^2 = 0.00; Chi^2 = 1988.34, df = 2 (P < 0.00001); I^2 = 100%				
Test for overall effect: Z = 109.30 (P < 0.00001)				
**4.2.3 Year 3**				
Davila-Seijo 2016	0.885	0.0004	33.6%	0.89 [0.88, 0.89]
Warren 2015	0.87	0.0002	33.6%	0.87 [0.87, 0.87]
Zweegers 2016	0.824	0.002	32.8%	0.82 [0.82, 0.83]
**Subtotal (95% CI)**			**100.0%**	**0.86 [0.85, 0.87]**
Heterogeneity: Tau^2 = 0.00; Chi^2 = 1720.49, df = 2 (P < 0.00001); I^2 = 100%				
Test for overall effect: Z = 115.48 (P < 0.00001)				
**4.2.4 Year 4**				
Davila-Seijo 2016	0.846	0.0005	33.8%	0.85 [0.85, 0.85]
Van den Reek 2016	0.84	0.0008	33.7%	0.84 [0.84, 0.84]
Zweegers 2016	0.811	0.0021	32.5%	0.81 [0.81, 0.82]
**Subtotal (95% CI)**			**100.0%**	**0.83 [0.82, 0.84]**
Heterogeneity: Tau^2 = 0.00; Chi^2 = 282.26, df = 2 (P < 0.00001); I^2 = 99%				
Test for overall effect: Z = 137.66 (P < 0.00001)				

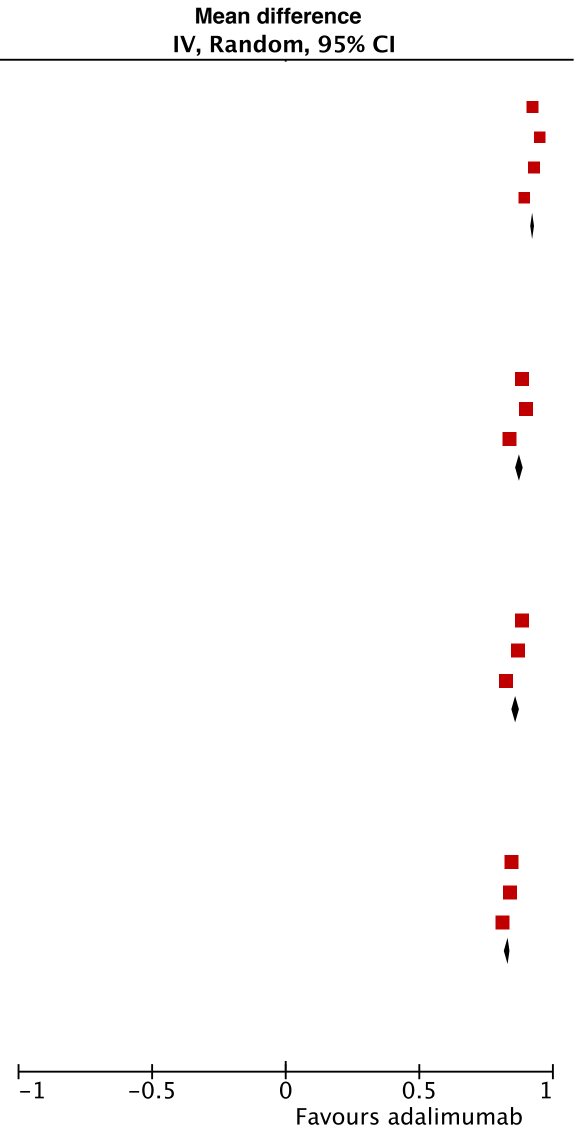

Test for subgroup differences: Chi^2 = 174.28, df = 3 (P < 0.00001), I^2 = 98.3%

Supplemetnary Figure S4(c): Forest plots of drug survival for discontinue due to adverse effects with infliximab.

Study or Subgroup	Risk Difference	SE	Weight	Mean difference IV, Random, 95% CI
**4.3.1 Year 1**				
Davila–Seijo 2016	0.846	0.0019	65.0%	0.85 [0.84, 0.85]
Warren 2015	0.84	0.0038	35.0%	0.84 [0.83, 0.85]
**Subtotal (95% CI)**			**100.0%**	**0.84 [0.84, 0.85]**
Heterogeneity: $Tau^2 = 0.00$; $Chi^2 = 1.99$, df = 1 (P = 0.16); $I^2 = 50\%$				
Test for overall effect: Z = 294.96 (P < 0.00001)				
**4.3.2 Year 2**				
Davila–Seijo 2016	0.808	0.0021	50.2%	0.81 [0.80, 0.81]
Warren 2015	0.74	0.0046	49.8%	0.74 [0.73, 0.75]
**Subtotal (95% CI)**			**100.0%**	**0.77 [0.71, 0.84]**
Heterogeneity: $Tau^2 = 0.00$; $Chi^2 = 180.84$, df = 1 (P < 0.00001); $I^2 = 99\%$				
Test for overall effect: Z = 22.77 (P < 0.00001)				
**4.3.3 Year 3**				
Davila–Seijo 2016	0.769	0.0022	50.0%	0.77 [0.76, 0.77]
Warren 2015	0.59	0.0051	50.0%	0.59 [0.58, 0.60]
**Subtotal (95% CI)**			**100.0%**	**0.68 [0.50, 0.85]**
Heterogeneity: $Tau^2 = 0.02$; $Chi^2 = 1038.61$, df = 1 (P < 0.00001); $I^2 = 100\%$				
Test for overall effect: Z = 7.59 (P < 0.00001)				
**4.3.4 Year 4**				
Davila–Seijo 2016	0.731	0.0024	100.0%	0.73 [0.73, 0.74]
**Subtotal (95% CI)**			**100.0%**	**0.73 [0.73, 0.74]**
Heterogeneity: Not applicable				
Test for overall effect: Z = 304.58 (P < 0.00001)				

Test for subgroup differences: $Chi^2 = 915.27$, df = 3 (P < 0.00001), $I^2 = 99.7\%$

Mean difference
IV, Random, 95% CI

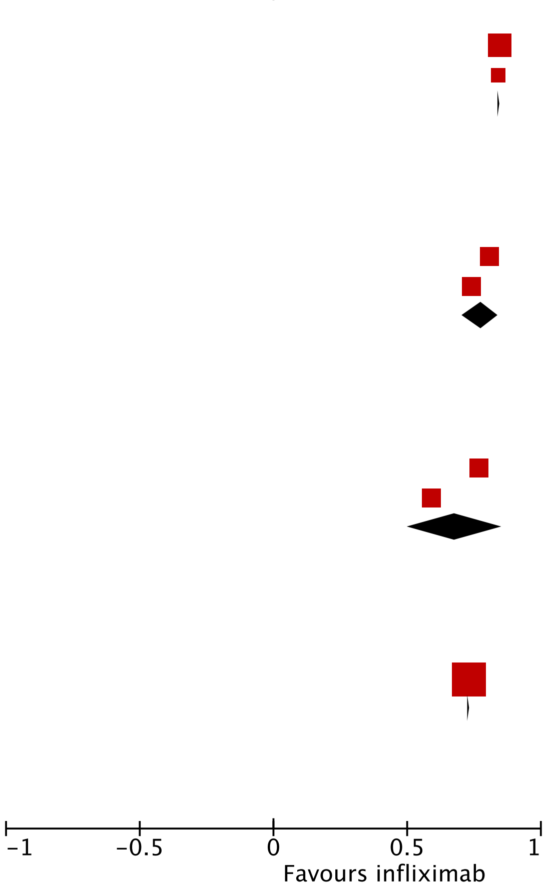

Supplemetnary Figure S4(d): Forest plots of drug survival for discontinue due to adverse effects with ustekinumab.

Study or Subgroup	Risk Difference	SE	Weight	Mean difference IV, Random, 95% CI
**4.4.1 Year 1**				
Davila-Seijo 2016	1	0		Not estimable
Van den Reek 2014b	0.95	0.0033	33.0%	0.95 [0.94, 0.96]
Warren 2015	0.96	0.0004	33.7%	0.96 [0.96, 0.96]
Zweegers 2016	0.919	0.0027	33.3%	0.92 [0.91, 0.92]
**Subtotal (95% CI)**			**100.0%**	**0.94 [0.92, 0.97]**
Heterogeneity: $Tau^2 = 0.00$; $Chi^2 = 233.17$, df = 2 ($P < 0.00001$); $I^2 = 99\%$				
Test for overall effect: Z = 71.31 ($P < 0.00001$)				
**4.4.2 Year 2**				
Davila-Seijo 2016	0.962	0.0004	33.5%	0.96 [0.96, 0.96]
Warren 2015	0.93	0.0006	33.5%	0.93 [0.93, 0.93]
Zweegers 2016	0.838	0.0036	33.0%	0.84 [0.83, 0.85]
**Subtotal (95% CI)**			**100.0%**	**0.91 [0.88, 0.94]**
Heterogeneity: $Tau^2 = 0.00$; $Chi^2 = 2966.20$, df = 2 ($P < 0.00001$); $I^2 = 100\%$				
Test for overall effect: Z = 57.51 ($P < 0.00001$)				
**4.4.3 Year 3**				
Davila-Seijo 2016	0.962	0.0004	33.4%	0.96 [0.96, 0.96]
Warren 2015	0.91	0.0006	33.4%	0.91 [0.91, 0.91]
Zweegers 2016	0.838	0.0036	33.2%	0.84 [0.83, 0.85]
**Subtotal (95% CI)**			**100.0%**	**0.90 [0.86, 0.95]**
Heterogeneity: $Tau^2 = 0.00$; $Chi^2 = 6092.37$, df = 2 ($P < 0.00001$); $I^2 = 100\%$				
Test for overall effect: Z = 39.88 ($P < 0.00001$)				
**4.4.4 Year 4**				
Davila-Seijo 2016	0.962	0.0004	50.0%	0.96 [0.96, 0.96]
Zweegers 2016	0.757	0.0042	50.0%	0.76 [0.75, 0.77]
**Subtotal (95% CI)**			**100.0%**	**0.86 [0.66, 1.06]**
Heterogeneity: $Tau^2 = 0.02$; $Chi^2 = 2360.96$, df = 1 ($P < 0.00001$); $I^2 = 100\%$				
Test for overall effect: Z = 8.39 ($P < 0.00001$)				

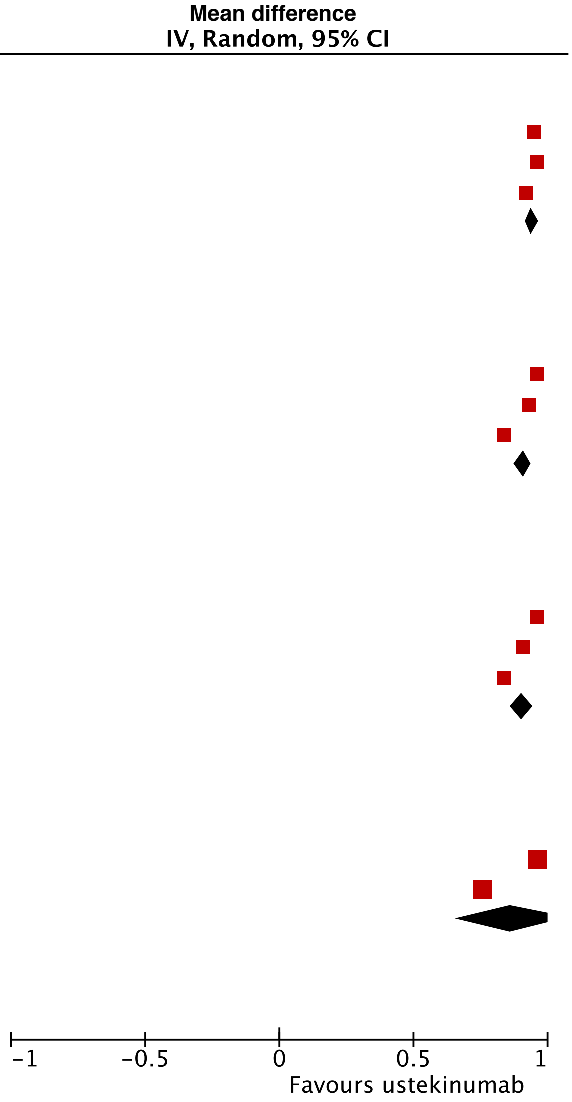

Test for subgroup differences: $Chi^2 = 4.02$, df = 3 ($P = 0.26$), $I^2 = 25.4\%$
